# Identification and characterization of CYP71 subclade cytochrome P450 enzymes involved in the biosynthesis of bitterness compounds in *Cichorium intybus*

**DOI:** 10.3389/fpls.2023.1200253

**Published:** 2023-06-22

**Authors:** Charlotte De Bruyn, Tom Ruttink, Elia Lacchini, Stephane Rombauts, Annelies Haegeman, Ellen De Keyser, Christof Van Poucke, Sandrien Desmet, Thomas B. Jacobs, Tom Eeckhaut, Alain Goossens, Katrijn Van Laere

**Affiliations:** ^1^ Plant Sciences Unit, Flanders Research Institute for Agriculture, Fisheries and Food (ILVO), Melle, Belgium; ^2^ Department of Plant Biotechnology and Bioinformatics, Ghent University, Ghent, Belgium; ^3^ Center for Plant Systems Biology, VIB, Ghent, Belgium; ^4^ Technology and Food Sciences Unit, Flanders Research Institute for Agriculture, Fisheries and Food (ILVO), Melle, Belgium; ^5^ Metabolomics Core, VIB, Ghent, Belgium

**Keywords:** chicory, sesquiterpene lactones, guaianolides, cytochrome P450, terpene synthase, jasmonate, *Nicotiana benthamiana*, CRISPR/Cas9 genome editing

## Abstract

Industrial chicory (*Cichorium intybus* var. *sativum*) and witloof (*C. intybus* var. *foliosum*) are crops with an important economic value, mainly cultivated for inulin production and as a leafy vegetable, respectively. Both crops are rich in nutritionally relevant specialized metabolites with beneficial effects for human health. However, their bitter taste, caused by the sesquiterpene lactones (SLs) produced in leaves and taproot, limits wider applications in the food industry. Changing the bitterness would thus create new opportunities with a great economic impact. Known genes encoding enzymes involved in the SL biosynthetic pathway are *GERMACRENE A SYNTHASE (GAS), GERMACRENE A OXIDASE (GAO)*, *COSTUNOLIDE SYNTHASE (COS)* and *KAUNIOLIDE SYNTHASE* (*KLS*). In this study, we integrated genome and transcriptome mining to further unravel SL biosynthesis. We found that *C. intybus* SL biosynthesis is controlled by the phytohormone methyl jasmonate (MeJA). Gene family annotation and MeJA inducibility enabled the pinpointing of candidate genes related with the SL biosynthetic pathway. We specifically focused on members of subclade CYP71 of the cytochrome P450 family. We verified the biochemical activity of 14 C*. intybus* CYP71 enzymes transiently produced in *Nicotiana benthamiana* and identified several functional paralogs for each of the *GAO*, *COS* and *KLS* genes, pointing to redundancy in and robustness of the SL biosynthetic pathway. Gene functionality was further analyzed using CRISPR/Cas9 genome editing in *C. intybus*. Metabolite profiling of mutant *C. intybus* lines demonstrated a successful reduction in SL metabolite production. Together, this study increases our insights into the *C. intybus* SL biosynthetic pathway and paves the way for the engineering of *C. intybus* bitterness.

## Introduction

The Asteraceae plant family comprises many economically relevant crops, including chicory, of which three cultivated groups are distinguished: root chicory, leaf chicory and witloof (Kiers et al., 2000; Barcaccia et al., 2016; Raulier et al., 2016). Root chicory, known as industrial chicory (*Cichorium intybus* var. *sativum*), is mainly grown for the extraction of inulin-type fructan carbohydrates, an important health-promoting dietary fiber (Chi et al., 2011), from the taproot. The industrial chicory root has further potential as a raw material to make a gluten-free, nutritionally beneficial chicory flour for use in the food industry. Within the leaf chicory group, Sugarloaf (*C. intybus* var. *porphyreum*), Radicchio (*C. intybus* var. *latifolium*), and Catalogne (*C. intybus* var. *sylvestre*) can be distinguished as subgroups, all consisting of leafy vegetables. Witloof (*C. intybus* var. *foliosum)*, also referred to as witlof, Belgian endive or chicon, composes a vegetable of tightly packed, etiolated leaves. *Cichorium intybus* crops are all characterized by a bitter taste (Drewnowski and Gomez-Carneros, 2000; Meyer and Blaauwhoed, 2009), attributed to the production of sesquiterpene lactones (SLs) as specialized metabolites. SLs also have a role in plant defense (Padilla-Gonzalez et al., 2016) and are responsible for many of the health benefits of chicory, including antifungal, anti-inflammatory, antitumor and anticytotoxic activity, and are therefore of economic importance (Barrero et al., 2000; Bischoff et al., 2004; Chadwick et al., 2013; Häkkinen et al., 2021; Meng et al., 2022). Better knowledge of the metabolic pathway and the functionality of the genes involved would enable modification of SL content and open new markets for *Cichorium* use and consumption.

**Figure 1:**
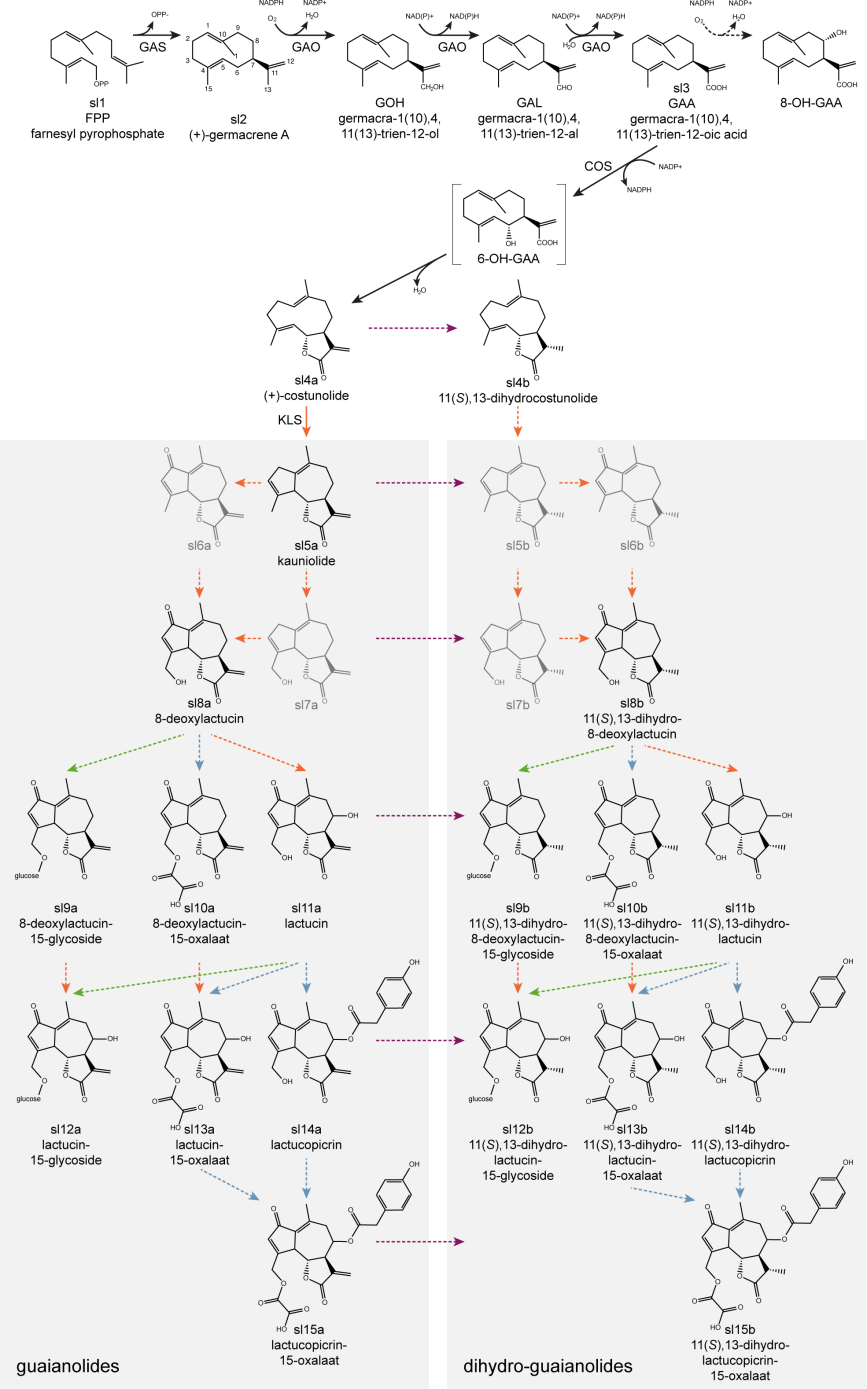
Proposed putative biosynthetic pathway for the formation of guaianolide SL metabolites. It starts with the cyclization of farnesyl pyrophosphate (FPP) to (+)-germacrene A by GERMACRENE A SYNTHASE (GAS), followed by the conversion into germacrene acid by GERMACRENE A OXIDASE (GAO), into (+)-costunolide by COSTUNOLIDE SYNTHASE (COS), and into kauniolide by KAUNIOLIDE SYNTHASE (KLS). Conversion of costunolide into 11(*S*),13-dihydrocostunolide occurs by an unknown enzyme. Both kauniolide and 11(*S*),13-dihydrocostunolide could be further converted into undetected intermediates (5b, 6a, 6b, 7a, 7b) necessary for subsequent conversions into guaianolide- (left) and dihydro-guaianolide- (right) SL metabolites, catalyzed by yet unknown enzymes. Arrows indicate putative conversions with enzymes belonging to oxidase (orange), reductase (purple), acyltransferase (blue) and glycosylase (green) gene families. Dashed arrows indicate putative pathway branches for which no enzymes have been identified yet. The point(s) of conversion from SL metabolites to dihydro-SL metabolites (on the right side of the purple dotted line) is (are) unknown.

The majority of SLs are thought to be derived from the germacranolides (De Kraker et al., 2001) with the guaianolides being the most important with regard to bitterness (De Kraker et al., 1998). The principal guaianolide SLs reported in *Cichorium* species are lactucin, 8-deoxylactucin, lactucopicrin and their derivatives, such as 11,13-dihydro-analogs (Van Beek et al., 1990). While multiple SL extraction, isolation, identification and quantification methods were developed (Ivanescu et al., 2015), a total of 16 guaianolide SL metabolites could be detected in leaf extracts of *C. intybus* using ultra-high performance liquid chromatography (UHPLC) – high-resolution mass spectrometry (HRMS) (Kips, 2017), and more recently one 12,8-guaianolide, four 12,6-guaianolides, and 16 analogs were isolated from *C*. *intybus* roots (Meng et al., 2022). Some guaianolide SL metabolites are present both in the free forms and as their oxalates or glycosides (bound to carbohydrates) (Ferioli et al., 2015; Kips, 2017) (**Figure 1**).

The biosynthetic pathway that forms bitter guaianolide SL metabolites has been studied in a number of plants of the Asteraceae family: *C. intybus* (Bouwmeester et al., 2002; Nguyen et al., 2010; Cankar et al., 2011; Liu et al., 2011; Bogdanović et al., 2019; Bogdanović et al., 2020; Cankar et al., 2021; Cankar et al., 2022), *C. endiva* (Zhang et al., 2022), *Helianthus annuus* and *Lactuca sativa* (Nguyen et al., 2010; Ikezawa et al., 2011), *Tanacetum parthenium* (Liu et al., 2014; Liu et al., 2018), *Artemisia annua* (Majdi et al., 2016)*, Saussurea lappa* and *Barnadesia spinosa* (Nguyen et al., 2010), among others. The biosynthesis starts with the cyclization of farnesyl pyrophosphate (FPP) by the terpene synthase (TPS) GERMACRENE A SYNTHASE (GAS) to form germacrene A (De Kraker et al., 1998; Bouwmeester et al., 2002) (**Figure 1**). Four paralogous *GERMACRENE A SYNTHASE* genes (*CiGAS*) have previously been identified in *C. intybus* (Bogdanović et al., 2019), of which two genes *CiGASl* and *CiGASs* were functionally characterized by Bouwmeester et al. (2002). In addition, CRISPR/Cas knock-out mutations in the four *CiGAS* paralogous genes reduce the SL metabolite levels (Cankar et al., 2021). Three-step hydroxylation/oxidation of the methyl group at C-12 of germacrene A by the cytochrome P450 (CYP) GERMACRENE A OXIDASE (GAO) then leads to the formation of germacrene A acid (Nguyen et al., 2010) (**Figure 1**). Subsequent hydroxylation at the C6- (or C8-) position of germacrene A acid by the CYP COSTUNOLIDE SYNTHASE (COS) produces an intermediate that undergoes spontaneous non-enzymatic lactonization to yield costunolide, the first SL metabolite (De Kraker et al., 2002; Ikezawa et al., 2011; Liu et al., 2011) (**Figure 1**). Two *CiGAO* genes and one *CiCOS* gene, isolated from *C. intybus*, have previously been functionally characterized (Nguyen et al., 2010; Cankar et al., 2011; Liu et al., 2011). The further conversion from the germacranolide SL substrate costunolide to guaianolide SLs was first established by the isolation and characterization of a CYP from feverfew (*T. parthenium*), KAUNIOLIDE SYNTHASE (KLS), which catalyzes the formation of the guaianolide kauniolide (Liu et al., 2018) (**Figure 1**). Recently, three *CiKLS* genes were also identified in chicory, and their catalytic activity was confirmed using a yeast microsome assay (Cankar et al., 2022). Inactivation of these *CiKLS* genes by CRISPR/Cas genome editing resulted in the interruption of SL biosynthesis in chicory leaves and taproots. Genes encoding enzymes involved further downstream of kauniolide have not been identified yet. Furthermore, alternative pathways leading to the formation of germacranolide and guaianolide SLs may exist, as it is currently unclear at which point in the pathway hydroxylation occurs to form the lactone, how dihydro-derivates are formed and at which point(s) glycosylation occurs (De Kraker et al., 1998; De Kraker et al., 2002).

Here, we describe a discovery workflow for genes involved in the SL biosynthetic pathway in *C. intybus*. First, a comprehensive genome-wide screen and gene family annotation were carried out and combined with phylogenetic and transcriptome analyses. We used an annotated reference genome of *C. intybus* var. *sativum* (Waegneer et al., 2023) to mine for all the corresponding genes of the two most important SL biosynthesis gene families, namely *TPS*s and *CYP*s. Using protein alignment and phylogenetic analysis, gene clades were delineated and clades were identified containing known members (*GAS*, *GAO*, *COS*, *KLS*) with a previously demonstrated biochemical activity in *C. intybus* or closely related plant species. Further, we performed a comprehensive transcriptome analysis of methyl jasmonate (MeJA)-treated samples of three species, *C. intybus* var. *sativum*, *C. intybus* var. *foliosum* and *L. sativa*, to confirm that SL biosynthesis is induced by MeJA and to further select *TPS* and *CYP* gene family members putatively involved in the SL biosynthetic pathway. Subsequent functional analysis by heterologous expression in *Nicotiana benthamiana* leaves established the enzymatic activity of several of the cloned *CYP* genes and determined their possible role in the SL biosynthetic pathway. CRISPR/Cas9 genome editing (De Bruyn et al., 2020) was used to create *GAO*, *COS*, and *KLS* mutants in *C*. *intybus* and provide insights into the *in planta* role of the *CYP* genes in the formation of the guaianolide SL metabolites.

## Materials and methods

### Gene identification and phylogenetic analysis

An in-house generated *C. intybus* var. *sativum* reference genome sequence (https://bioinformatics.psb.ugent.be/orcae/overview/Cicin) (Waegneer et al., 2023) was used for genome-wide identification of gene family members of the selected *TPS* and *CYP* gene families. First, the corresponding homology groups (HOMgroups) with all known orthologs of multiple species were extracted from the PLAZA dicots 4.0 database (https://bioinformatics.psb.ugent.be/plaza) (Van Bel et al., 2018). The coding DNA sequences (CDS) and protein sequences were used as queries to identify the *C. intybus* homologs using BLASTn and BLASTp searches (e-value <1e-80), respectively, against the CDS and protein sequences of all 53,507 predicted *C. intybus* genes. ORCAE (Sterck et al., 2012) was used for manual curation of the structural gene annotation. Each predicted gene was manually curated, using available supporting data in ORCAE (such as RNA-sequencing (RNA-seq) read alignments, splicing sites, gapped alignments of *de novo* assembled transcripts, and blast hits of orthologs from closely related species). Predicted *C. intybus* proteins were further validated via multiple protein sequence alignment (MUSCLE) and predicted *TPS* and *CYP C. intybus* genes leading to proteins smaller than 350 amino acids or without a C-terminus were classified as pseudogenes. A tBLASTx was performed between the CDS of *C. intybus* and *L. sativa* (V8, 2016-01-20, id28333, https://lgr.genomecenter.ucdavis.edu/) (Reyes-Chin-Wo et al., 2017) in both directions (e-value <1e-80) to identify the corresponding *L. sativa* orthologs (as *L. sativa* is the closest relative to *Cichorium* containing also a fully sequenced and annotated genome and produces guaianolide SL metabolites). *L. sativa* genes were not manually curated but genes leading to proteins smaller than 350 amino acids were classified as pseudogenes. Proteins encoded by *C. intybus* and *L. sativa* genes (excluding the pseudogenes), were used for phylogenetic analysis (UPGMA tree) in Geneious 10.2.6 (http://www.geneious.com). The *C. intybus* CYP protein sequences were further classified by David R. Nelson (University of Tennessee, USA).

### RNA-sequencing and differentially expressed genes analysis

*C. intybus* var. *foliosum* ‘Topmodel’ and ‘Van Tongelen’, *C. intybus* var. *sativum* ‘OBOE’ and ‘VL70’ and *L. sativa* ‘ZORBA’ seeds were sterilized in 70% EtOH (1 min) and 1.5% NaOCl + 0.02% Tween20 (15 min). Eight sterilized seeds were transferred to sterile plastic containers (145 mm x 100 mm x 60 mm; Eco2nv, Zottegem, Belgium) filled with growth medium (4.4 g.L^-1^ Murashige and Skoog (MS) + vitamins (Murashige and Skoog, 1962), 20 g.L^-1^ sucrose, 7 g.L^-1^ agar, pH 5.8). Seeds were topped with an autoclaved 145 mm x 100 mm 100 µM mesh (Prosep bvba, Zaventem, Belgium) and containers were kept in a growth chamber for 2 weeks at 23 ± 2°C under a 16-h/8-h (light/dark) photoperiod at 40 µmol.m^-2^.s^-1^ photosynthetic active radiation (PAR). After two weeks of growth, the number of seedlings was reduced to four well-grown seedlings per container and the mesh containing the seedlings was transferred to mock growth medium (4.4 g.L^-1^ MS + vitamins, 20 g.L^-1^ sucrose, 7 g.L^-1^ agar, 0.02% Tween20, pH 5.8) or growth medium supplemented with 100 µM MeJA. After 2 h, 6 h or 24 h of treatment, the four seedlings in each container were pooled and frozen in liquid nitrogen. A total of 120 samples (five plant varieties, two treatments, three time points, four biological replicates each) were harvested.

Reverse transcription quantitative polymerase chain reaction (RT-qPCR) was performed to validate MeJA induction of *CiGASl* (AF497999.1), *CiGASs* (AF498000.1), *CiGAO* (GU256644.1) and *CiCOS* (JF816041.1) candidate genes and to select the most suitable samples for RNA-seq. RNA was extracted from 150 to 200 mg ground plant material, using the 3% CTAB RNA extraction protocol (Luypaert et al., 2017). RNA extraction, quantification and RT-qPCR were performed as previously described (De Keyser et al. (2020). Eleven reference genes were selected for industrial chicory and witloof (Delporte et al., 2015) and ten for lettuce (Borowski et al., 2014; Sgamma et al., 2016). The geNorm (Vandesompele et al., 2002) module in qbase+ (Hellemans et al., 2007) (CellCarta) was used for reference gene selection. RT-qPCR with the reference genes was performed in a LightCycler480 (Roche). RT-qPCR primers targeting *CiGASl*, *CiGASs*, *CiGAO* and *CiCOS* were developed (**Supplementary Table 1**) to amplify at least one paralogous gene copy. RT-qPCR analysis was done on the entire sample set, all no-RT samples, including no template controls (NTC) using both target and selected reference genes [*TIP41* and *PP2AA2* for industrial chicory and witloof (M-value = 0.124; CV-value = 0.043) and *TIP41* and *PP2AA3* for lettuce (M-value = 0.423; CV-value = 0.146)].

A total of 18 RNA samples were selected (three species, i.e. *C*. *intybus* var. intybus, *C*. *intybus* var. foliosum, *L. sativa*, three biological replicates each per MeJA treatment versus mock) for RNA-seq analysis. Libraries were prepared and sequenced by the VIB Nucleomics Core facility (Leuven, Belgium) with SE-75 reads on an Illumina NextSeq500 instrument (NCBI SRA Bioproject PRJNA738883). Adapters and low quality 3’ ends were trimmed from the RNA-seq reads (with a quality threshold of 20) using Cutadapt (Martin, 2011). Next, reads were dereplicated using prinseq-lite (Schmieder and Edwards, 2011). Industrial chicory and witloof reads were mapped on the CDS of an assembled and annotated reference genome of *C. intybus* var. *sativum* (Waegneer et al., 2023), while lettuce reads were mapped on the CDS of the annotated reference genome of *L. sativa* (Reyes-Chin-Wo et al., 2017), using BWA-MEM (Li and Durbin, 2009) with default settings. Mapped reads were sorted using SAMtools (Li et al., 2009) and reads with a mapping quality below 20 were discarded. Subsequent data analysis was done in R v.3.4.3 (R Core Team, 2017) using the package edgeR (Robinson et al., 2010). Genes that occurred in less than two samples with less than four counts per million reads mapped (CPM) were discarded. Normalization to scale the raw library sizes was performed using the trimmed mean of M-values (to the reference) as proposed by Robinson and Oshlack (2010). To identify differentially expressed genes (DEGs), a negative binomial generalized log-linear model was fitted to the read counts for each gene and subsequently likelihood ratio tests were performed between the MeJA treatment and the mock per cultivar. Thresholds for further filtering of the DEGs were set at minimum 2-fold expression change (log2-fold change of 1) and an adjusted p-value (Benjamini-Hochberg multiple testing correction) cutoff of 0.05 was used to be further considered as statistically significant DEGs between MeJA and mock treatment. A heatmap was constructed visualizing the gene expression ratio (log2-fold change) of MeJA- over mock-treated samples of industrial chicory, witloof and lettuce.

### Cloning of *C*. *intybus* candidate genes and *N. benthamiana* leaf infiltration

Full-length CDS (flCDS) of the *CiGAO*, *CiCOS*, *CiKLS* and *CYP71BZ* candidate genes were amplified from cDNA with Q5 High Fidelity Polymerase (New England Biolabs) using gene-specific primers (**Supplementary Table 2**). PCR amplification products were used in an additional PCR with Q5 High Fidelity Polymerase using the gene-specific primers flanked by AttB sites for Gateway cloning. The PCR amplification products were run on a 1.5% agarose gel, extracted and purified with the GeneJET Gel Extraction Kit (Thermo Fisher Scientific). Previously identified *CiGASs* (AF498000.1) (Bouwmeester et al., 2002), *CiGAO* (GU256644.1) (Nguyen et al., 2010), *CiCOS* (JF816041.1) (Liu et al., 2011) and *TpKLS* (MF197559.1) (Liu et al., 2018) gene coding sequences were synthesized by Twist Bioscience (San Francisco, US). All amplified or synthesized sequences were cloned into the Gateway donor vector pDONR207 (Invitrogen) using Gateway™ BP clonase™ (Thermo Fisher Scientific) and sequence-verified by Sanger sequencing (Eurofins) prior to insertion into the *N. benthamiana* binary expression vector pEAQ-HT-DEST1 (Sainsbury et al., 2009) using Gateway™ LR clonase™ (Thermo Fisher Scientific).

The recombinant *N. benthamiana* expression vectors were individually transformed into the *Agrobacterium tumefaciens* strain C58C1, carrying the pMP90 helper plasmid, by electroporation. Transformed *A. tumefaciens* were grown and used for infiltration in *N. benthamiana* (Moses et al., 2015). Each vector was infiltrated to the abaxial side of three fully expanded leaves of 3- to 4-week-old *N. benthamiana* plants using 1-mL syringes. Infiltration with *A. tumefaciens* containing an empty pEAQ-HT-DEST1 vector was used as a negative control. The agroinfiltrated plants were incubated for 3 days in a plant growth chamber maintained at 25°C in a 14-h/10-h (light/dark) photoperiod.

### Metabolite extraction and GC-MS analysis

Agroinfiltrated *N. benthamiana* leaves were harvested and ground to a fine powder in liquid nitrogen. Around 100 mg of leaf material was used for extraction of the SL metabolites. Leaf material was first vortexed with 1 mL of hexane for 10 min, followed by centrifuging for 15 min at 3,000 rpm. The resulting organic extract was evaporated to dryness under vacuum. For GC-MS analysis, the residue obtained from metabolite extraction was trimethylsilylated using 10 µL of pyridine and 50 µL of N-methyl-N-(trimethylsilyl)trifluoroacetamine. GC-MS analysis was performed on a 7890B GC system and a 7250 GC/QTOF (Agilent). Analyses were performed on a VF-5ms capillary column (30m x 0.25 mm x 0.25 µm; Varian CP9013; Agilent) at a constant helium flow of 1.2 mL/min, using a 1-µL aliquot, which was injected in splitless mode. After injection, the oven was held at 70°C for 2 min, ramped to 210°C at a rate of 5°C/min, held at 210°C for 5 min, ramped to 320°C at a rate of 20°C/min, held at 320°C for 5 min, and finally cooled to 70°C at a rate of 50°C/min at the end of the run. The injector, the MS transfer line, the MS ion source, and the quadrupole were set to 280°C, 280°C, 230°C and 150°C, respectively. Full electron ion (EI)-MS spectra were generated for each sample by scanning the m/z range of 50 to 800 with a solvent delay of 15 min. Electron ionization energy was 70 eV. For relative quantification, the peak areas were integrated using Agilent Masshunter Quantitative Analysis software (version 10.0). Identification of costunolide in the agroinfiltrated *N. benthamiana* leaves was performed by comparison with the retention time and mass spectrum of a costunolide standard (Sigma-Aldrich).

### CRISPR/Cas9 genome editing in *Cichorium*

**Figure 2:**
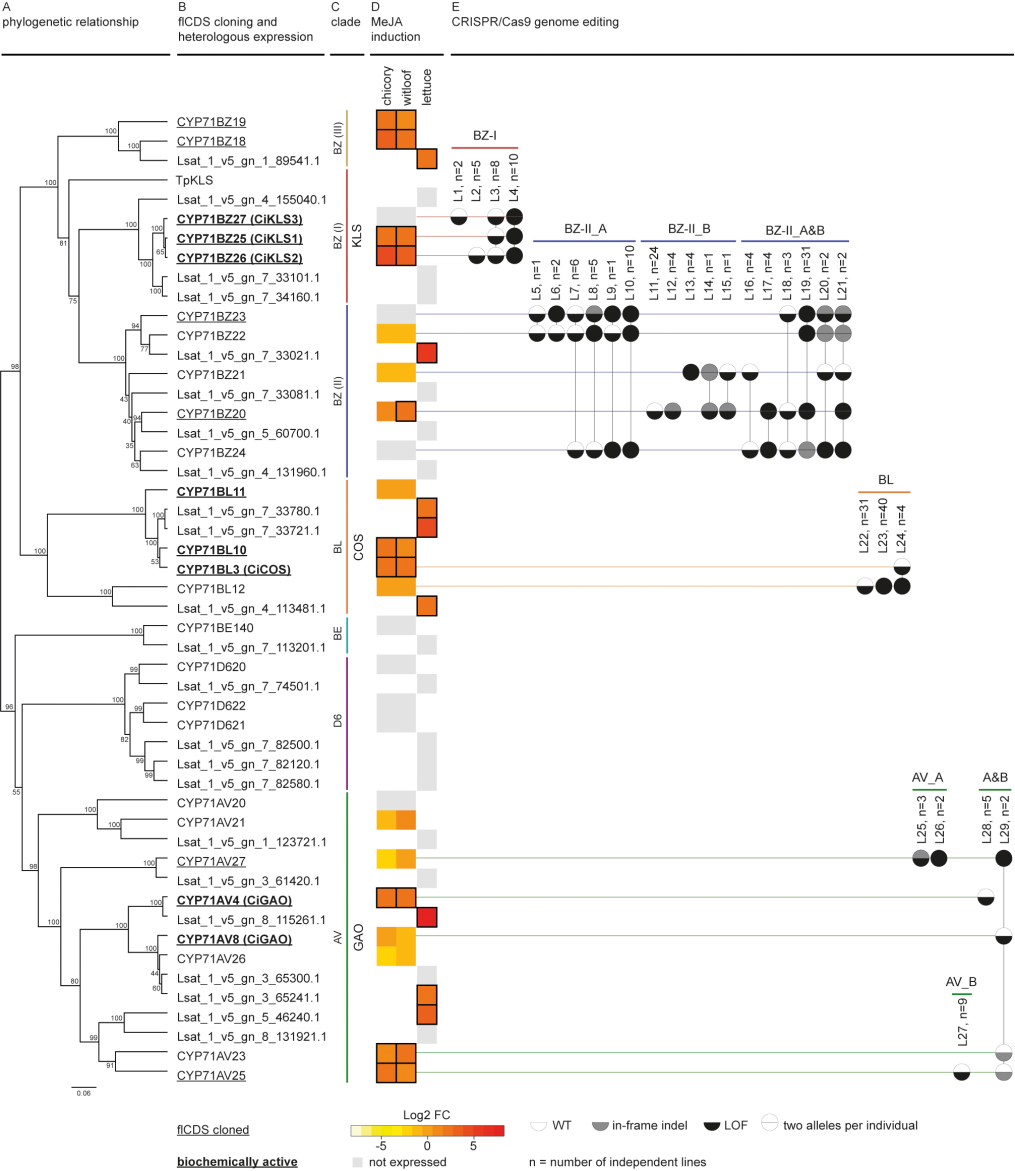
Discovery workflow of *C. intybus CYP71* genes. **(A)** Phylogenetic analysis of *C. intybus* and corresponding *L. sativa* orthologous *CYP71* genes. The phylogenetic tree was inferred from the protein alignment using the UPGMA method. Bootstrap values (shown at the branching points) are based on 1,000 replicates. **(B)** Full-length (fl)CDS were cloned from 14 genes (underlined), and functionally tested by heterologous expression in *N. benthamiana* (bold=biochemically active). KLS from *T. parthenium* (*TpKLS*) (AXG24152.1) was included for phylogenetic analysis. *CYP71AV4/CYP71AV8* and *CYP71BL3* were previously identified as functional *CiGAO* (ADF43080.1) and *CiCOS* (AEG79727.1), respectively. Also *CiKLS1*, *CiKLS2*, *CiKLS3* were previously described as functional. **(C)** The various clades are indicated by vertical lines for GAO (green), COS (orange) and KLS (red). **(D)** Heatmap of gene expression response to MeJA treatment (log2-fold change in gene expression versus mock treatment as measured via RNA-seq) in industrial chicory, witloof and lettuce. Gray boxes indicate non-expressed genes while MeJA-inducible genes with log2-fold change ≥ 1 are marked with a black outline. **(E)** CRISPR/Cas9 genome editing was applied by targeting groups of genes (GAO_A, GAO_B, COS, KLS, BZ-II_A, BZ-II_B, and BZ-III). Mutants (listed by mutation type in **Supplementary Table 5**) are shown as two alleles per gene per circle, colored by wild type (WT), in-frame indel, or loss-of-function (LOF). LOF types are labeled L1-L29 and aggregated number of plants (n) are given.

Plant material of industrial chicory *C. intybus* var. *sativum* ‘L9001’ was provided by COSUCRA (Belgium), and witloof *C. intybus* var. *foliosum* ‘Van Hamme’ was provided by Nationale Proeftuin voor Witloof (Herent, Belgium) and subcultured *in vitro* as previously described (De Bruyn et al. (2020). To target multiple putative paralogous *CiGAO*, *CiCOS, CiKLS* or *CYP71BZ* genes, the identified *CiGAO*, *CiCOS*, *CiKLS* and *CYP71BZ* clades were further divided into groups GAO_A, GAO_B, COS, KLS, BZ-II_A, BZ-II_B and BZ-III using nucleotide alignments and phylogenetic clustering (**Supplementary Figure 1**). Multiple gRNAs were designed to target the putative paralogous SL biosynthesis genes of each subclade (see **Supplementary Table 3** for the corresponding primers). CRISPR/Cas9 vector construction, protoplast isolation, transfection and regeneration were performed as previously described (De Bruyn et al. (2020) with minor changes. Protoplasts were cotransfected with up to four CRISPR/Cas9 vectors, each containing a unique gRNA (grouped per subclade), using a total of 20 µg vector. The CRISPR/Cas9 gene target sequences and primers used for HiPlex amplicon sequencing are shown in **Supplementary Figure 1** for each gene (see **Supplementary Table 4** for the corresponding primer sequences). HiPlex amplicon sequencing, ploidy level and mutation analyses were performed as described in De Bruyn et al. (2020). Mutant types were defined as carriers of unique mutations (*i.e.*, a discrete set of non-reference haplotype alleles identified by *SMAP haplotype-window* (Schaumont et al., 2022)). Mutant types were further aggregated based on the predicted consequences of the mutation(s) on the encoded protein functionality. By assuming loss of function (LOF) in case of out-of-frame indels or large in-frame indels (>9 amino acids), M types were grouped into L types if the functional consequences were similar, despite small differences in the actual mutated sequence. L types were defined by the homozygous or heterozygous LOF state per locus, for a given set of mutated genes (**Figure 2** and **Supplementary Table 5**).

### Metabolite extraction and UHPLC-HRMS analysis

For metabolite profiling of CRISPR/Cas9 genome-edited plants and wild-type control plants, for each plant, around 3 g of inner leaves were freshly harvested after between 2 and 6 months of growth and used for three technical replicates. Only for regenerant M3 and the two M6 genotypes material could be harvested for only one analysis without replicates. Each plant sample was immediately frozen and ground in liquid nitrogen, freeze-dried and stored under vacuum conditions at -20°C till analysis. Given that chicory bitterness is mostly attributed to the SLs downstream of compounds sl7a and sl7b (**Figure 1**), we performed metabolite profiling of the genome-edited plants only by UHPLC-HRMS analysis. To this end, extraction, separation and detection were done according to the method developed by Kips (2017). All compounds (sl08a: deoxylactucin, sl08b: dihydrodeoxylactucin, sl09a: deoxylactucin glycoside, sl09b: dihydrodeoxylactucin glycoside, sl10a: deoxylactucin oxalate, sl10b: dihydrodeoxylactucin oxalate, sl11a: lactucin, sl11b: dihydrolactucin, sl12a: lactucin glycoside, sl12b: dihydrolactucin glycoside, sl13a: lactucin oxalate, sl13b: dihydrolactucin oxalate, sl14a: lactucopicrin, sl14b: dihydrolactucopicrin, sl15a: lactucopicrin oxalate and sl15b: dihydrolactucopicrin oxalate) were identified based on the accurate mass and fragmentation pattern and reported as relative peak areas (area compound/area internal standard (santonine, Sigma-Aldrich)). Four compounds were quantified with reference standards: lactucin (sl11a), dihydrolactucin (sl11b), lactucopicrin (sl14a) and dihydrolactucopicrin (sl14b) (all from Extrasynthese, Genay, France), while no standards were available for the other compounds. Data recording was achieved with MassLynx™ (v.4.1) while the integration was performed with TargetLynx™ (v. 4.1) (Waters). Data analysis of metabolite profiles was done in R v.4.0.4 (R Core Team, 2017). Mean and standard error values were calculated for each mutation type of the relative peak areas of all 16 guaianolide SL metabolites extracted from the plants containing mutations in (multiple) paralogous *CiGAO*, *CiCOS* or *CiKLS* genes. Significant SL metabolite changes between the wild-type plants and the mutants were analyzed using a Pairwise Wilcoxon Rank Sum Test (p < 0.05).

## Results

### Genome-wide annotation of *C. intybus* TPS and CYP families

Given their reported involvement in SL biosynthesis, our *C. intybus* var. *sativum* reference genome sequence (https://bioinformatics.psb.ugent.be/orcae/overview/Cicin) (Waegneer et al., 2023) was used for genome-wide identification of members from the *TPS* and *CYP* gene families. In total, 447 C*. intybus* annotated genes were manually curated, respectively 51 *TPS*s and 396 *CYP*s, of which 87 (19%) were classified as pseudogenes. Identification of the corresponding *L. sativa* orthologs resulted in 52 *TPS*s and 391 *CYP*s, with 42 (9%) pseudogenes. In summary, excluding pseudogenes, 40 *TPS* and 320 *CYP* gene family members were identified in *C. intybus* and 47 *TPS*s and 354 *CYP*s in *L. sativa*. Both *C. intybus* and *L. sativa* protein models were used for the creation of the TPS (**Supplementary Figure 2**) and CYP (**Supplementary Figure 3**) phylogenetic trees and clade classification.

### Identification of MeJA-inducible genes by RNA-seq

**Figure 3:**
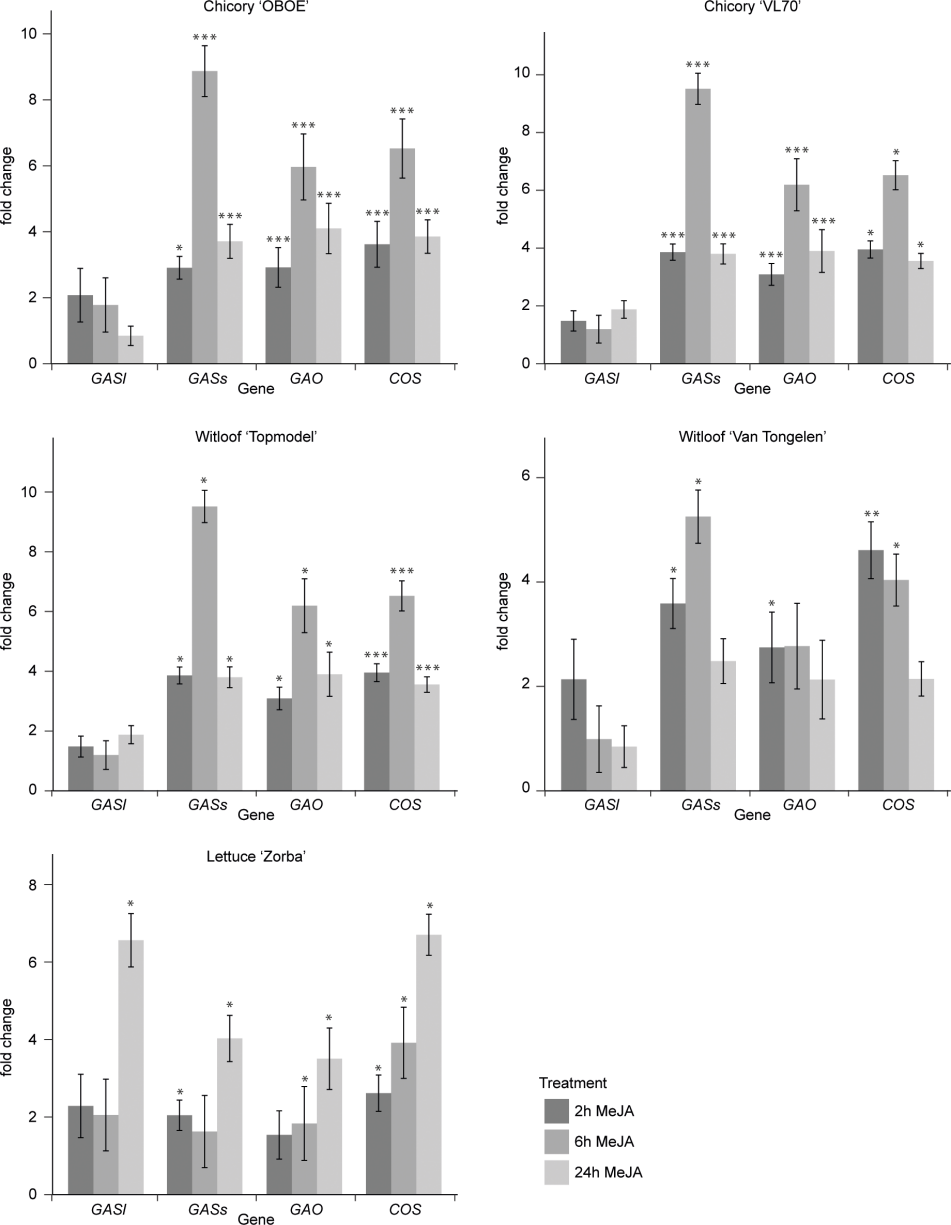
Fold changes of SL biosynthesis gene expression. Steady-state levels of transcripts corresponding to *CiGASl*, *CiGASs*, *CiGAO* (*CYP71AV4*) and *CiCOS* (*CYP71B3*)) were determined by RT-qPCR in seedlings of five plant varieties after 2 h, 6 h and 24 h of mock or MeJA treatment. Columns reflect the increase in transcript levels caused by MeJA treatment. The values in the *y*-axis correspond to the mean ± SE (n = 4) of the ratio of the transcript levels in MeJA-treated samples versus those in mock-treated samples at each timepoint. Statistical differences relative to the respective mock treatments were determined by Kruskal-Wallis rank sum test at p<0.05, using Pairwise Wilcoxon rank sum test (*p<0.05, **p<0.01 and ***p<0.001.).

Jasmonates (JAs) are widely known as elicitors of plant specialized metabolism, including many terpene biosynthetic pathways (Wasternack and Strnad, 2019; Lacchini and Goossens, 2020; Nguyen et al., 2022). Notably, transcript profiling studies have demonstrated that treatment with JA or MeJA induces the expression of genes encoding enzymes involved in the biosynthesis of artemisinin, the SL from the medicinal plant *Artemisia annua* (Yu et al., 2012). Yet, potential responsiveness to JAs has to the best of our knowledge not been investigated for guaianolide SL production in *C. intybus* or any other species. Therefore, we performed a comprehensive transcriptome analysis of seedlings of three species, *C. intybus* var. *sativum*, *C. intybus* var. *foliosum* and *L. sativa*, treated with MeJA over 24 h. First, we assessed the MeJA responsiveness of the known SL biosynthetic pathway genes *CiGASl*, *CiGASs*, *CiGAO*, and *CiCOS* by RT-qPCR analysis. This analysis confirmed the MeJA responsiveness of the SL pathway in all three species (**Figure 3**). As such, it became plausible that MeJA inducibility could be relevant to select candidate *CYP* gene family members potentially involved in *C. intybus* SL biosynthesis. Strongest upregulation was observed after a 6-h MeJA treatment in all three species. Thus, samples of industrial chicory ‘OBOE’, witloof ‘Topmodel’ and lettuce ‘ZORBA’, after 6 h of mock or MeJA treatment were used for subsequent RNA-seq analysis. The industrial chicory and witloof reads were mapped to the CDS of *C. intybus* var. *sativum*, containing 53,507 annotated genes, of which 38% and 37% were expressed in industrial chicory and witloof, respectively. The lettuce reads were mapped to the CDS of *L. sativa*, containing 37,829 annotated genes (Reyes-Chin-Wo et al., 2017), of which 48% were expressed (**Supplementary Table 6**). Hereof, 3,384 industrial chicory genes, 2,696 witloof genes and 1,395 lettuce genes were significantly differentially expressed between MeJA and mock treatment (p_adj_<0.05) (**Supplementary Table 6**). Comparison between industrial chicory and witloof gene sets identified a total of 1,930 genes that were commonly differentially expressed upon MeJA treatment. As anticipated, these included known SL biosynthesis genes. In industrial chicory, witloof, and lettuce, the number of downregulated genes (MeJA vs. mock) was 1,489, 1,076 and 586, respectively, and the number of upregulated genes (MeJA vs. mock) was 1,895, 1,620 and 809, respectively (**Supplementary Table 6**).

### Integration of MeJA inducibility and phylogenetic analysis for the selection of candidate SL biosynthesis genes

A total of 40 and 47 *TPS* gene family members were identified in *C. intybus* and *L. sativa*, respectively, of which 15 industrial chicory, 14 witloof and 16 lettuce genes were MeJA-inducible (**Supplementary Table 6**; **Supplementary Figure 2**). Protein alignment of all *TPS* genes showed that the previously described *CiGAS-L*, the tandem duplicated genes *CiGAS-S1* and *CiGAS-S2*, and *CiGAS-S3* (Bouwmeester et al., 2002; Bogdanović et al., 2019; Cankar et al., 2021) corresponded to *cicin09g10370*, *cicin03g50310* and *cicin03g50280*, and *cicin06g42820*, respectively, thus identifying the clade with functional CiGAS proteins (**Supplementary Figure 2**). Two additional *CiGAS* paralogs belonging to the same clade were identified: *cicin06g42850* and *cicin02g48520* (**Supplementary Figure 2** and **Supplementary Figure 4**). The six paralogs were located on four different chromosomes, with two tandem duplicated genes on CiChr3 and two tandem duplicated genes on CiChr6. Of these, *cicin03g50310*, *cicin03g50280* and *cicin06g42820* were MeJA-inducible in both industrial chicory and witloof (**Supplementary Figure 2**). In the same clade, four lettuce orthologs were identified, of which only *Lsat_1_v5_gn_8_116340.1* was MeJA-inducible. Based on the protein alignment and phylogenetic clustering of the *TPS* genes with the previously described *CiGAS* genes, a total of six genes in the genome of *C. intybus* could potentially encode GAS enzymes, more specifically *cicin03g50280, cicin03g50310, cicin06g42850, cicin06g42820, cicin09g10370*, and *cicin02g48520* (**Supplementary Figure 2**; GAS clade indicated by the blue vertical bar).

A total of 320 and 354 genes were identified in the *CYP* superfamily in *C. intybus* and *L. sativa*, respectively, of which 49 industrial chicory, 51 witloof and 33 lettuce genes were MeJA-inducible (**Supplementary Table 6** **;** **Supplementary Figure 3**). Phylogenetic analysis identified the *CYP71* clade with the previously described *CiGAO*, *CiCOS* and *CiKLS* genes (Liu et al., 2018; Cankar et al., 2022), the clade on which we further focused in this study. This *CYP71* clade contains a total of 26 C*. intybus* and 23 *L. sativa* genes (**Figures 2A, B**), of which 9 industrial chicory, 10 witloof and 8 lettuce genes were MeJA-inducible (**Figure 2D**). Phylogenetic analysis further identified three subclades that each contained multiple putative paralogs of *CiGAO, CiCOS* and *CiKLS* genes (**Figure 2C**).

Two functional *CiGAO* genes (*CYP71AV4* and *CYP71AV8*) were previously described in *C. intybus* (Nguyen et al., 2010; Cankar et al., 2011; Liu et al., 2011). Our analysis revealed eight potential paralogs in the *CYP71AV CiGAO* clade (**Figures 2A–C** and **Supplementary Figure 4**): *CYP71AV4* located on CiChr3, *CYP71AV27* located on CiChr7, tandem duplicated genes *CYP71AV8* and *CYP71AV26* located on CiChr7, *CYP71AV23* and *CYP71AV25* located on CiChr4, and the slightly more distantly related *CYP71AV20* and *CYP71AV21* located on CiChr2. *CYP71AV4*, *CYP71AV23*, and *CYP71AV25* were MeJA-inducible in both industrial chicory and witloof. In the same clade, seven lettuce orthologs were identified, of which three genes (*Lsat_1_v5_gn_8_115261.1*, *Lsat_1_v5_gn_3_65241.1* and *Lsat_1_v5_gn_5_46240.1*) were MeJA-inducible (**Figure 2D**).

Four potential paralogs were identified in the *CYP71BL CiCOS* clade: *CYP71BL11*, *CYP71BL10*, *CYP71BL3* and *CYP71BL12* (**Figures 2A–C** and **Supplementary Figure 4**), which were all located on CiChr5 in proximity of each other. *CYP71BL3* revealed 99.8% nucleotide identity (one SNP difference) to the previously described functional *CiCOS* gene (Liu et al., 2011). *CYP71BL10* and *CYP71BL3* were MeJA-inducible in both industrial chicory and witloof (**Figure 2D**). Within the same clade, three lettuce orthologs were identified, which were all MeJA-inducible (*Lsat_1_v5_gn_7_33780.1*, *Lsat_1_v5_gn_7_33721.1* and *Lsat_1_v5_gn_4_113481.1*).

The *CYP71BZ* clade containing *CiKLS* and *TpKLS* revealed a total of ten potential paralogs in *C. intybus* (**Figures 2A–C** and **Supplementary Figure 4**), divided in three subclades (here named I to III). The three tandem duplicated genes, *CYP71BZ25, CYP71BZ26*, and *CYP71BZ27* of subclade I, corresponding to *CiKLS1*, *CiKLS2*, and *CiKLS3* (Cankar et al., 2022), respectively, were located on CiChr5. Of these, *CiKLS1* and *CiKLS2* were MeJA-inducible, while their lettuce orthologs (*Lsat_1_v5_gn_4_155040.1, Lsat_1_v5_gn_7_33101.1, Lsat_1_v5_gn_7_34160.1*) were not expressed. *CYP71BZ* subclade II contained five genes; *CYP71BZ22*, *CYP71BZ23* and *CYP71BZ24* were tandem duplicated genes located on CiChr5, while *CYP71BZ21* and *CYP71BZ20* were tandem duplicated genes located on CiChr4, and only *CYP71BZ20* was MeJA-inducible in witloof. Within that subclade, a total of four lettuce orthologs were identified, of which only *Lsat_1_v5_gn_7_33021.1* was MeJA-inducible. In subclade III, the tandem duplicated genes *CYP81BZ19* and *CYP71BZ18* located on CiChr2 were both MeJA-inducible, as was their lettuce ortholog (*Lsat_1_v5_gn_1_89541.1*).

Finally, two other *CYP71* subclades were identified: *CYP71BE* with one gene of chicory and lettuce each (*CYP71BE140*, *Lsat_1_v5_gn_7_113201.1*), and *CYP71D6* with three genes of chicory (*CYP71D620, CYP71D621*, and *CYP71D622*), and four orthologs of lettuce (*Lsat_1_v5_gn_7_74501.1*, *Lsat_1_v5_gn_7_82500.1*, *Lsat_1_v5_gn_7_82120.1, Lsat_1_v5_gn_7_82580.1*), respectively, but none of these genes were MeJA-inducible.

### Reconstruction of costunolide production in *N. benthamiana*

**Figure 4:**
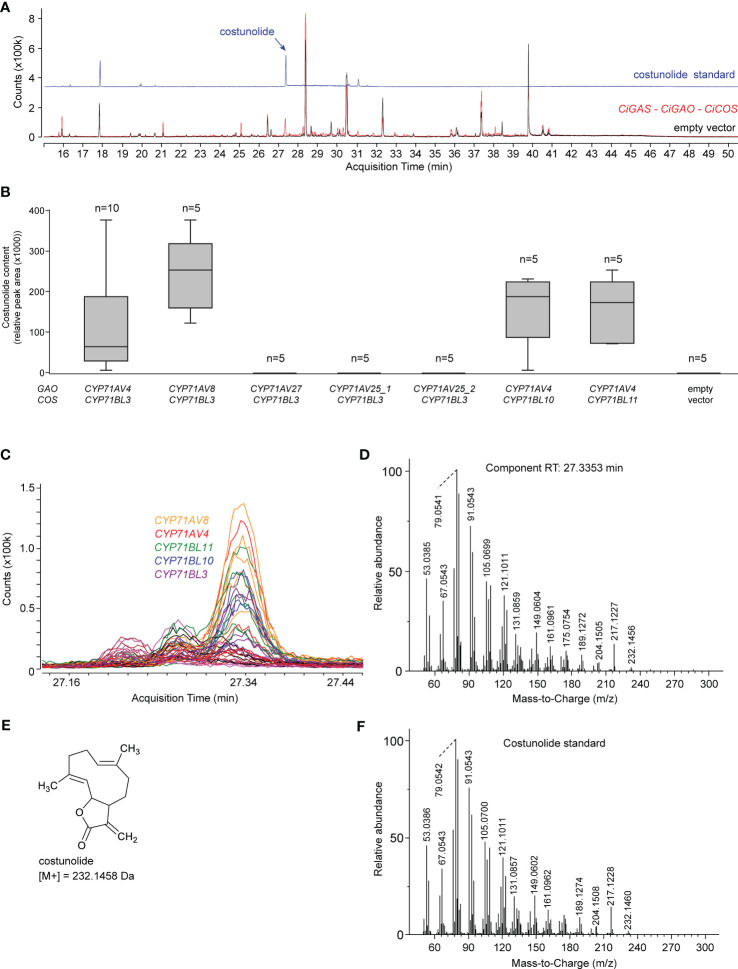
Costunolide production in agroinfiltrated *N. benthamiana* leaves. **(A)** Overlay of the EIC GC-MS chromatograms of the costunolide standard (blue) and *N. benthamiana* extracts co-agroinfiltrated with *CiGASs*, *CYP71AV4* (*CiGAO*) and *CYP71BL3 (CiCOS)* (red) or empty vector controls (black). The peak at ±27.3 min (indicated with a black arrow) corresponds to costunolide. **(B)** Boxplot of costunolide production (relative peak area) in *N. benthamiana* leaves (n = 3) co-agroinfiltrated with *CiGASs* and various combinations of potential *CiGAO* and *CiCOS* genes, including *CYP71AV4* (*CiGAO*), *CYP71AV8* or paralogous candidate *CiGAO* genes (*CYP71AV27*, *CYP71AV25*), and *CYP71BL3 (CiCOS)* or paralogous candidate *CiCOS* genes (*CYP71BL10*, *CYP71BL11*). Labels _1, _2, etc. indicate different allelic variants of the corresponding *CYP71* genes cloned from the different *C. intybus* varieties used. **(C)** Overlay of the EIC (232.15 ± 0.05 Da; 27.1 – 27.5 min) GC-MS chromatograms of all samples (three replicates of all assays in **Supplementary Table 10**). The peak at 27.34 min corresponds to the blue peak in panel **(B)** The assays displayed indicate which agroinfiltrated *N. benthamiana* extracts produce this peak. The peak was not present in all other assays, indicated by all colored horizontal lines. **(D)** Deconvoluted EI-MS spectrum of the peak eluting at 27.34 min. **(E)** Structure of costunolide with the corresponding molecular weight. **(F)** Deconvoluted EI-MS spectrum of the costunolide standard.

To investigate which of the candidate *CYP71* paralogous genes described above are involved in SL biosynthesis, we first reconstructed the *C. intybus* SL biosynthesis pathway up to costunolide in *N. benthamiana* leaves by agroinfiltration (**Figure 4**). The SL-specific candidate genes were co-expressed with a gene encoding a truncated, feedback-free version of the 3-HYDROXY-3-METHYLGLUTARYL-COA REDUCTASE (tHMGR) enzyme (Reed et al., 2017), to boost production of the precursor of all sesquiterpenes. First, the successfully cloned flCDS of four putative paralogous *CiGAO* genes were transformed in *N. benthamiana* leaves by co-agroinfiltration with *CiGASs* (*cicin03g50280*; also described as *CiGAS-S2* in (Cankar et al., 2021)) and *CiCOS* (*CYP71BL3*) to assess GAO functionality and/or relative catalytic efficiency (**Supplementary Table 7**). Co-agroinfiltration of *CiGASs, CiGAO* and *CiCOS* was used as a positive control for the production of costunolide, as described previously (Liu et al., 2018). Costunolide was detected in *N. benthamiana* leaves transformed with *CiGAO* (*CYP71AV4*) and *CYP71AV8*, but not *CYP71AV27* or *CYP71AV25* (**Figures 4B, C**). Notably, the activity of *CYP71AV8*, which is non-MeJA-inducible (**Figure 2D**), was relatively higher than that of *CYP71AV4*.

The successfully cloned flCDS of three putative paralogous *CiCOS* genes were then transformed in *N. benthamiana* leaves by co-agroinfiltration with *CiGASs* (*cicin03g50280*) and *CiGAO* (*CYP71AV4*) (**Supplementary Table 7**). Costunolide could be detected in *N. benthamiana* leaves transformed with each of these *CiCOS* paralogs (**Figures 4B, C**). As with *CiGAO* (*CYP71AV4*), this included MeJA-inducible (*CYP71BL3* and *CYP71BL10*) and non-MeJA-inducible (*CYP71BL11*) genes (**Figure 2D**). No marked differences in the catalytic performance of the three paralogs was apparent.

In conclusion, a total of two paralogous *CiGAO* genes (*CYP71AV8* and *CYP71AV4*) and three paralogous *CiCOS* genes (*CYP71BL10*, *CYP71BL11* and *CYP71BL3*) displayed catalytic activity in the synthetic costunolide biosynthetic pathway in *N. benthamiana*.

### Reconstruction of kauniolide production in *N. benthamiana*

**Figure 5:**
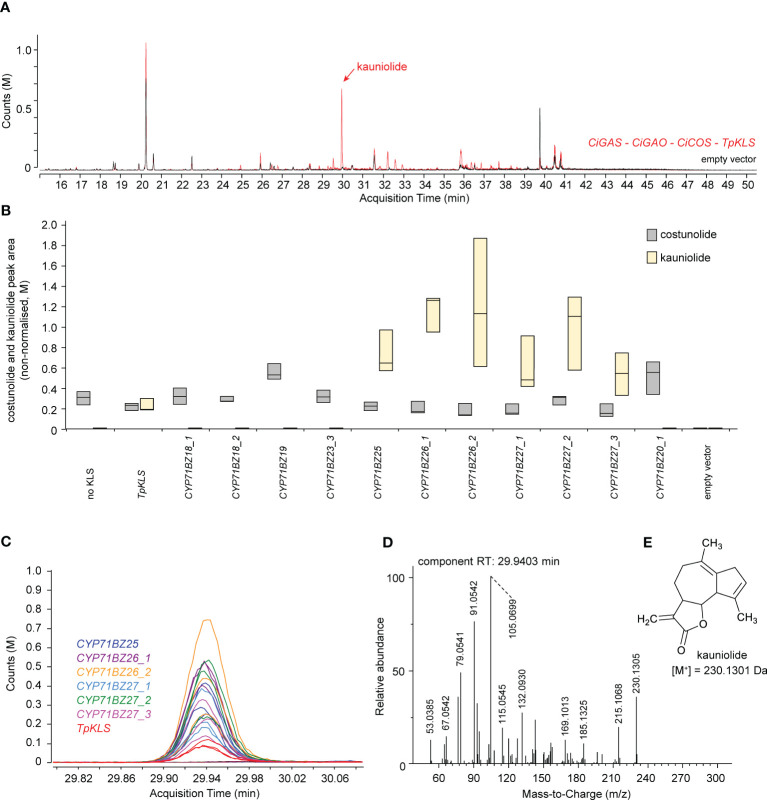
Kauniolide production in agroinfiltrated *N. benthamiana* leaves. **(A)** Overlay of the EIC GC-MS chromatogram of *N. benthamiana* extracts co-agroinfiltrated with *CiGASs*, *CYP71AV4* (*CiGAO*), *CYP71BL3 (CiCOS), and TpKLS* (red chromatogram) or empty vector controls (black chromatogram). The red peak at ±30 min (indicated with a black arrow) is present in the *N. benthamiana* extracts co-agroinfiltrated with both *CYP71BL3* and *TpKLS* and absent in the *N. benthamiana* extracts agroinfiltrated with only *CYP71BL3*. **(B)** Boxplot of kauniolide production (relative peak area) in *N. benthamiana* leaves (n = 5) co-agroinfiltrated with *CiGASs*, *CYP71AV4* (*CiGAO*), *CYP71BL3 (CiCOS)*, and differential potential *KLS* genes, including *TpKLS, CiKLS* or paralogous candidate *CiKLS* genes. Labels _1, _2, etc. indicate different allelic variants of the corresponding *CYP71* genes cloned from the different *C. intybus* varieties used. **(C)** Overlay of the EIC (230.13 ± 0.05 Da; 29.8 – 30.1 min) GC-MS chromatograms of all samples (three replicates of all assays in **Supplementary Table 10**). The peak at 29.94 min corresponds to the red peak in panel **(B)** The assays displayed indicate which agroinfiltrated *N. benthamiana* extracts produce this peak. The peak was not present in all other assays, indicated by all colored horizontal lines. **(D)** Deconvoluted EI-MS spectrum of the peak eluting at 29.94 min. **(E)** Structure of kauniolide with the corresponding molecular weight.

Successfully cloned flCDS of seven *CiKLS* subclade genes were transformed in *N. benthamiana* leaves by co-agroinfiltration with *CiGASs, CiGAO* (*CYP71AV4*) and *CiCOS* (*CYP71BL3*) (**Supplementary Table 8**). Co-agroinfiltration of *CiGASs, CiGAO* and *CiCOS* with *TpKLS* was used as positive control for the potential production of kauniolide (**Figure 5**), as previously described (Liu et al., 2018). Kauniolide was detected in *N. benthamiana* leaves transformed with *TpKLS* as well as with *CiKLS1* (*CYP71BZ25*), *CiKLS2* (*CYP71BZ26*) and *CiKLS3* (*CYP71BZ27*) (**Figures 5A–C**). No kauniolide production was observed in any of the other combinations (*CYP71BZ18* and *CYP71BZ19* (subclade III); *CYP71BZ20* and *CYP71BZ23* (subclade II)). As with *CiGAO* and *CiCOS*, the functional *CiKLS* gene set included MeJA-inducible (*CYP71BZ25* and *CYP71BZ26*) and non-MeJA-inducible (*CYP71BZ27*) genes (**Figure 2D**).

In conclusion, a total of three paralogous *CiKLS* genes displayed catalytic activity in the synthetic kauniolide biosynthetic pathway in *N. benthamiana*. Thus, these results correlated well with those published by Cankar et al. (2022), who demonstrated the KLS activity of these three genes in a yeast microsome assay.

### Other CYP71BZ subclade members do not metabolize costunolide or kauniolide in *N. benthamiana*

To investigate whether any of the hitherto non-functional *CYP71BZ* genes of subclades II and III could act in SL biosynthesis, the flCDS of all remaining successfully cloned, non-functional *CYP71* genes were transformed in *N. benthamiana* leaves by co-agroinfiltration with *CiGASs, CiGAO*, *CiCOS* and *CiKLS2* (**Supplementary Table 9**). None of the tested combinations led to further conversion of kauniolide to putative downstream SLs, suggesting that these *CYP71* family members may not be involved in SL biosynthesis, accept costunolide or kauniolide as a substrate, or be expressed or functional in *N. benthamiana*.

### CRISPR/Cas9-induced mutations in the *CYP71* gene family

CRISPR/Cas9 genome editing was used to simultaneously target multiple *CYP71* genes in protoplasts of *C. intybus*, followed by regeneration of plants. For this experiment, the identified *CiGAO*, *CiCOS* and *CiKLS* (sub)clades were further divided into groups (GAO_A, GAO_B, COS, KLS, BZ-II_A, BZ-II_B and BZ-III; **Figure 2E**) and multiple gRNAs were designed to target the putative paralogous SL biosynthesis genes within each group (**Supplementary Figure 1**). A series of CRISPR/Cas9 protoplast co-transfection and regeneration experiments using up to four CRISPR/Cas9 vectors (combined per group) were performed to create a broad spectrum of plant genotypes containing different (LOF) mutations in multiple genes. A total of 939 regenerated plants were acclimatized to the greenhouse and characterized by HiPlex amplicon sequencing at all target loci, 46 plants targeted in the *CiGAO* paralogous candidate genes, 109 plants targeted in the *CiCOS* paralogous candidate genes, and 784 plants targeted in the *CiKLS or CYP71BZ* paralogous candidate genes., respectively. Mutation analysis revealed 225 plants with at least one mutated allele in one SL biosynthesis candidate gene (**Figure 2E**), which were grouped into 46 unique mutation types (**Supplementary Table 5**), and further aggregated into LOF types. An overall plant mutation frequency of 23.9% was obtained, creating a broad spectrum of mutants. Ploidy levels were determined for 368 acclimatized plants, including 46 control plants and 322 CRISPR/Cas9-edited plants, resulting in 48.1% diploid plants and 51.9% tetraploid plants. However, the tetraploid mutated plants did not contain more complex editing patterns (no more than two unique alleles detected per locus), suggesting that the ploidy change occurred after the mutation was induced.

### SL metabolite profiling of SL biosynthesis pathway mutants

36 CRISPR/Cas9 mutants, each containing mutations in one or more paralogous *CYP71* genes, were sampled for SL metabolite profiling (4 *GAO* mutants, 13 *COS* mutants, 21 *KLS* or *CYP71BZ* mutants; **Supplementary Table 5**), corresponding to different 23 mutation types. The production of 14 out of 16 SL metabolites was successfully eliminated in one mutant type (M6) (**Supplementary Table 10**), containing a homozygous LOF mutation in the three paralogous *CiKLS* genes, which were functionally characterized here and in Cankar et al. (2022). These results confirm that the three paralogous *CiKLS* genes are indeed important SL biosynthesis genes for the production of the guaianolide SL metabolites in chicory. Unfortunately, the plants with LOF alleles in the three *CiKLS* genes were slow-growing and not persistent, making it impossible to include biological and technical replicates. Therefore, no statistical analysis could be performed on the SL metabolite values of these mutants compared to wild-type control plants. Besides mutation type M6, no other mutation type that could be analyzed showed such a consistent noticeable modulation in SL levels (**Supplementary Table 10**). None of the mutant lines with LOF alleles for the genes encoding the CYP71 proteins that were non-functional in *N. benthamiana* (subclade BZ-II) showed any detectable alteration in SL levels, further indicating that these genes are not involved in SL biosynthesis in *C. intybus*. Nonetheless, it should be noted that for genes from subclade BZ-III in the *CYP71* phylogenetic tree (e.g. *CYP71BZ18* and *CYP71BZ19*) that were non-functional in *N*. *benthamiana*, we did not generate homozygous LOF mutations, hence their possible involvement cannot be excluded unambiguously yet.

It should further be noted that most SL-profiled plants (also the wild type) were tetraploid plants. Comparing the SL metabolite content of the four quantitatively measurable (ng/mg dry weight) SL metabolites, lactucin, lactucopicrin, dihydrolactucin and dihydrolactucopicrin, in the analyzed diploid and tetraploid plants (**Supplementary Table 10**; see M3, M46 and WT) showed an SL metabolite concentration increase in these tetraploid plants, suggesting that the plant’s ploidy level has an influence on SL metabolite production.

## Discussion

Modifying the bitterness of *C. intybus* could open new markets for this crop in addition to the existing inulin market. Not only reducing the bitterness in *Cichorium* can be of value, but also increasing the production of SL metabolites can improve market options, such as the extraction of bioactive SLs as natural compounds for the pharmaceutical and cosmetic industries (Häkkinen et al., 2021; Matos et al., 2021; Rolnik and Olas, 2021). Although there is some natural variation in the SL content among *Cichorium* species (Foster et al., 2011; Testone et al., 2016), variants with a considerably low SL content have not yet been identified. Better knowledge of the pathway and the functionality of the genes involved would open opportunities for modification of SL biosynthesis.

### Genome-wide gene family annotation to identify genes with potential SL biosynthetic activity

Delineation of the putative biochemical functions involved in the SL biosynthetic pathway based on a hypothetical SL pathway connecting the conversions of FPP to the detectable guaianolide SL metabolites (Kips, 2017) and our current knowledge of this pathway drove us to select two specific enzyme families, TPS and CYP. The functional diversity of the TPS enzymes underlies the generation of diverse terpenoids across the plant kingdom (Karunanithi and Zerbe, 2019). In this study, we found 40 genes encoding TPSs in the genome of *C. intybus*, including *cicin03g50310*, *cicin03g50280*, *cicin06g42820*, and *cicin09g10370*, which correspond to the previously described *CiGAS-S1*, *CiGAS-S2*, *CiGAS-S3* and *CiGAS-L*, respectively (Bouwmeester et al., 2002; Bogdanović et al., 2019; Cankar et al., 2021). The CYP superfamily is one of the largest enzyme families in plants and has also been widely identified in animals, fungi, protists, archaea, bacteria, insects and viruses (Xu et al., 2015). In this study, 320 genes were found to potentially encode functional CYPs in the genome of *C. intybus*, including *CYP71AV4*/*CYP71AV8* and *CYP71BL3*, which were previously functionally characterized as *CiGAO* and *CiCOS*, respectively (Nguyen et al., 2010; Cankar et al., 2011; Liu et al., 2011), as well as the three recently identified *CiKLS* genes (Cankar et al., 2022). Several phylogenetic clades were identified in the two gene families, and anchored to *CiGAS*, *CiGAO*, *CiCOS*, and *CiKLS*. Notably, each clade contains multiple putative paralogous genes, often derived from tandem duplication, which generally display high levels of amino-acid sequence similarity, suggesting similar protein folding structures and putatively conserved biochemical activities (e.g. *CiKLS1*, *CiKLS2*, *CiKLS3*). Such a comprehensive genome-wide overview of paralogs and the underlying mechanisms that drive gene family expansion and gene evolution (including non-, sub- and neo-functionalization) is important in the frame of pathway elucidation. For instance, it defines how many and which genes (redundantly) encode enzymes at each subsequent step of the pathway, which regulatory elements are shared or unique across paralogs (leading to co-expression networks and/or control of pathway activity by alternative regulatory factors or signaling pathways), and whether knockouts, e.g. by genome editing, will lead to observable phenotypes (*e.g.* will LOF mutations be required in multiple genes to overcome functional redundancy).

### Functional analysis of *CYP71* genes via heterologous expression in *N. benthamiana* and CRISPR/Cas9 genome editing in *C. intybus* reveal multiple functional paralogs of *GAO*, *COS* and *KLS*

Given the reported importance of members of the *CYP71* gene family in *C. intybus* SL biosynthesis, we performed an in-depth characterization of this gene family. First, the *CYP71* genes were functionally characterized using an *N*. *benthamiana* heterologous expression assay. In addition to confirming the two previously described *CiGAO* genes (Nguyen et al., 2010; Cankar et al., 2011), one *CiCOS* (Liu et al., 2011), and the three *CiKLS* genes (Cankar et al., 2022), two additional functional *CiCOS* genes were identified. Conversely, no biochemical activity was observed for *CYP71AV25*, *CYP71AV27*, *CYP71BZ18*, *CYP71BZ19*, *CYP71BZ20*, and *CYP71BZ23* in this system, suggesting that these candidate genes may either be non-functional in the guaianolide SL biosynthetic pathway, or may act further downstream.

The role of the putative paralogous *CYP71* genes in SL biosynthesis was also analyzed in *C. intybus* by the creation of LOF mutants. A broad spectrum of mutants was created containing mutations in single or multiple paralogous *CiGAO*, *CiCOS*, *CiKLS* and *CYP71BZ* genes and their guaianolide SL metabolite levels were determined. Because many of the *CiGAO*, *CiCOS*, *CiKLS* or *CYP71BZ* mutated plant genotypes only contained one heterozygous LOF allele in one paralog of a specific SL biosynthesis gene (*CiGAO*, *CiCOS*, *CiKLS* or *CYP71BZ*), the mutation effect on guaianolide SL metabolite quantities and composition was in most cases most likely masked due to the presence of at least one functional allele and/or functional paralog. There was one notable exception: mutation type M6 that contained a LOF mutation in the three paralogous *CiKLS* genes eliminated the accumulation of nearly all SL metabolites detectable in wild-type plants. These results further underscore that the enzymes encoded by these three *CiKLS* genes are necessary for *in planta* SL biosynthesis, more particularly for the production of the kauniolide intermediate, thereby corroborating similar observations reported by Cankar et al. (2022). Furthermore, our results also suggest that successful LOF mutations in all alleles of multiple paralogs of a specific SL biosynthesis gene are required to significantly decrease guaianolide SL metabolite production levels.

In this study, we also showed that the *C. intybus* SL pathway is controlled by the phytohormone MeJA, a known elicitor of plant specialized metabolism. Interestingly, when we integrated the outcome of the comprehensive RNA-seq analysis of MeJA-elicited *C. intybus* seedlings with that of the phylogenetic and functional analysis of the *CYP71* gene family, we discovered that all the functional clades, i.e. *CiGAO, CiCOS*, and *CiKLS*, contained both MeJA-inducible and non-MeJA-inducible paralogs, suggesting the existence of multiple regulatory routes controlling *C. intybus* SL biosynthesis. Furthermore, MeJA inducibility was often conserved between industrial chicory and witloof orthologs and even some orthologous *L. sativa* genes, indicating that the control of SL biosynthesis by this hormone evolved before the species diversification. Conversely, some genes were only MeJA-inducible in one *C. intybus* variety (industrial chicory or witloof) or *L. sativa*, which appoints them as plausible candidates for contributing to SL differentiation between these species (Testone et al., 2016).

### Engineering chicory bitterness

Reducing SL metabolite production in C. *intybus* has also been achieved via targeting of the *CiGAS* genes. Bogdanović et al. (2020) used gene silencing via artificial microRNAs to downregulate the expression of *CiGASs* and *CiGASl* genes. This approach resulted in only partial gene silencing, leading to variable levels of SL metabolite production. It should also be noted that the authors only evaluated the guaianolide oxalate SL metabolites, because these SL metabolites are often present in high amounts in wild-type *Cichorium* plants (Sessa et al., 2000). Cankar et al. (2021) used CRISPR/Cas9 genome editing to target the four previously identified *CiGAS* paralogs (Bogdanović et al., 2019), resulting in a significant decrease of all six measured SL metabolites but with a corresponding increase of squalene and phenolic compounds. Because FPP is the precursor for sesquiterpene and sterol biosynthesis, it is not surprising that inactivation of *CiGAS* leads to changes in the metabolic flux from FPP to other compounds. Indeed, accumulation of FPP has been shown upon silencing of the *amorpha-4,11-diene synthase* genes in *A*. *annua* (Catania et al., 2018) while silencing of terpene synthase genes (*TPS9* and *TPS12*) in tomato led to the upregulation of several genes involved in flavonoid biosynthesis (Coppola et al., 2018). The changes in these metabolic pathways with FPP as precursor should be considered when evaluating the bitter taste in plants containing mutations in the guaianolide SL biosynthesis genes.

Van Beek et al. (1990) were the first to show that the bitterness in *Cichorium* plants is related to their guaianolide SL metabolite concentration and composition. However, not all guaianolide SL metabolites that can be successfully measured today have been considered regarding bitterness perception. Almost all studies measuring guaianolide SL metabolite profiles focus on measuring six guaianolide SL metabolites: lactucin, 8-deoxylactucin, lactucopicrin, 11(S),13-dihydro-8-deoxylactucin, 11(S),13-dihydrolactucin and 11(S),13-dihydrolactucopicrin (Van Beek et al., 1990; Sessa et al., 2000; Foster et al., 2011; Beharav et al., 2015; D’antuono et al., 2016; Yanagisawa and Misaka, 2021). The oxalate and glycoside metabolite forms of guaianolide SL are not taken into account (Kips (2017), resulting in the unknown perception of their bitter taste. Still, previous studies analyzing these six guaianolide SL metabolites have shown that 11(S),13-dihydrolactucopicrin is perceived as extremely bitter, while lactucopicrin, 8-deoxylactucin, 11(S),13-dihydro-8-deoxylactucin, 11(S),13-dihydrolactucin and lactucin are perceived as less bitter. Furthermore, previous research on the quantitative analysis of these six guaianolide SL metabolites showed that the bitterness responses were dose-dependent and that the concentration at which the bitterness is perceived is different for each metabolite (Yanagisawa and Misaka, 2021). Moreover, the perception of bitterness seems not only to be related to the guaianolide SL metabolite concentrations and composition. A previous study strongly correlated glucose and sucrose concentrations in industrial chicory leaves with crunchiness and bitterness, while fructose concentrations were correlated with sweetness (François et al., 2008). Another study indicated that the balance between different SL metabolites and phenolic compounds affects the bitterness in a rather complex manner (D’antuono et al., 2016). Therefore, SL metabolites are possibly not the only metabolites that should be considered when studying bitterness in plant tissues. Cellular assays and human sensory tests can be used to evaluate the link between SL metabolite concentration and composition, and bitterness (Yanagisawa and Misaka, 2021).

## Data availability statement

The datasets presented in this study can be found in online repositories. The names of the repository/repositories and accession number(s) can be found below: https://www.ncbi.nlm.nih.gov/bioproject/; PRJNA738883.

## Author contributions

CDB, AG, TR, and KVL conceived and designed the research and supervised the experiments; CDB, SR, CVP, SD, and EL performed the experiments; SR designed and supervised genomic DNA extraction and sequencing, did assemblies and gene prediction; CDB, TR, SR, AH, CVP, SD, EL, TJ, TE, AG, and KVL designed the experiments and analyzed the data; CDB, TR, AG, and KVL wrote the article. All authors contributed to the article and approved the submitted version.
