# Supplementary material for: Identification and characterization of CYP71 subclade cytochrome P450 enzymes involved in the biosynthesis of bitterness compounds in Cichorium intybus
Source: Front Plant Sci. 2023 Jun 22;14:1200253. doi: 10.3389/fpls.2023.1200253 (PMC10324620; doi:10.3389/fpls.2023.1200253)
Supplement: Supplementary file 1 [file DataSheet_1.pdf]

Supplementary information

Identification and Characterization of CYP71 Subclade Cytochrome P450 Enzymes Involved in the Biosynthesis of Bitterness Compounds in *Cichorium intybus*

Charlotte De Bruyn, Tom Ruttink, Elia Lacchini, Stephane Rombauts, Annelies Haegeman, Ellen De Keyser, Christof Van Poucke, Thomas Jacobs, Tom Eeckhaut, Alain Goossens, Katrijn Van Laere

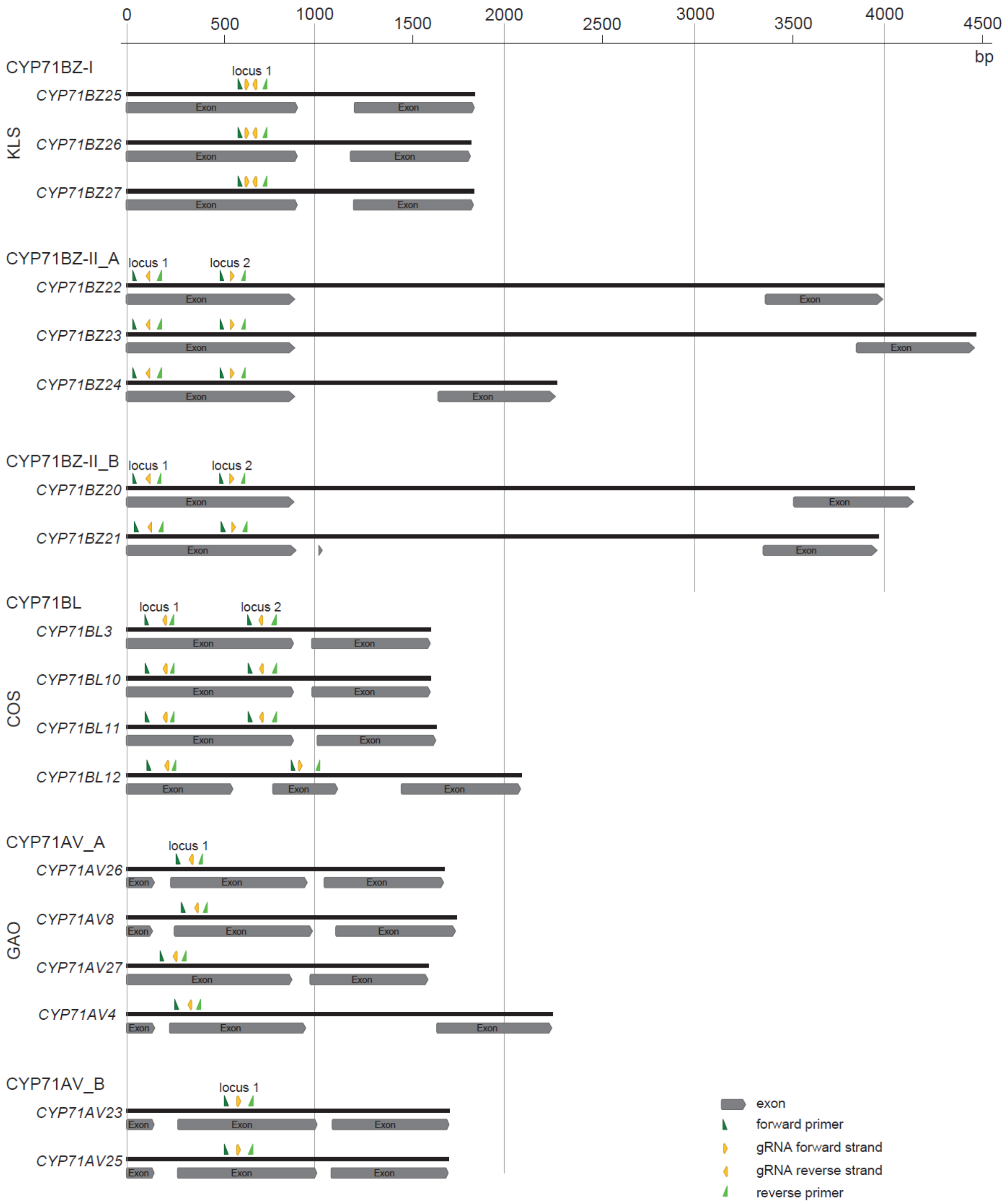

Supplementary Figure 1. Nucleotide alignments of groups of putative paralogous *CiGAO*, *CiCOS*, *CiKLS* and *CYP71BZ* genes (**Figure 2E**), visualizing CRISPR/Cas9 20-bp gRNA target sites and primers for HiPlex amplicon sequencing. Annotations: gray = exon; each colored ⬟ = unique forward 20-bp gRNA target site and each colored ⬟ = unique reverse 20-bp gRNA target site; dark green = forward primer; light green = reverse primer.

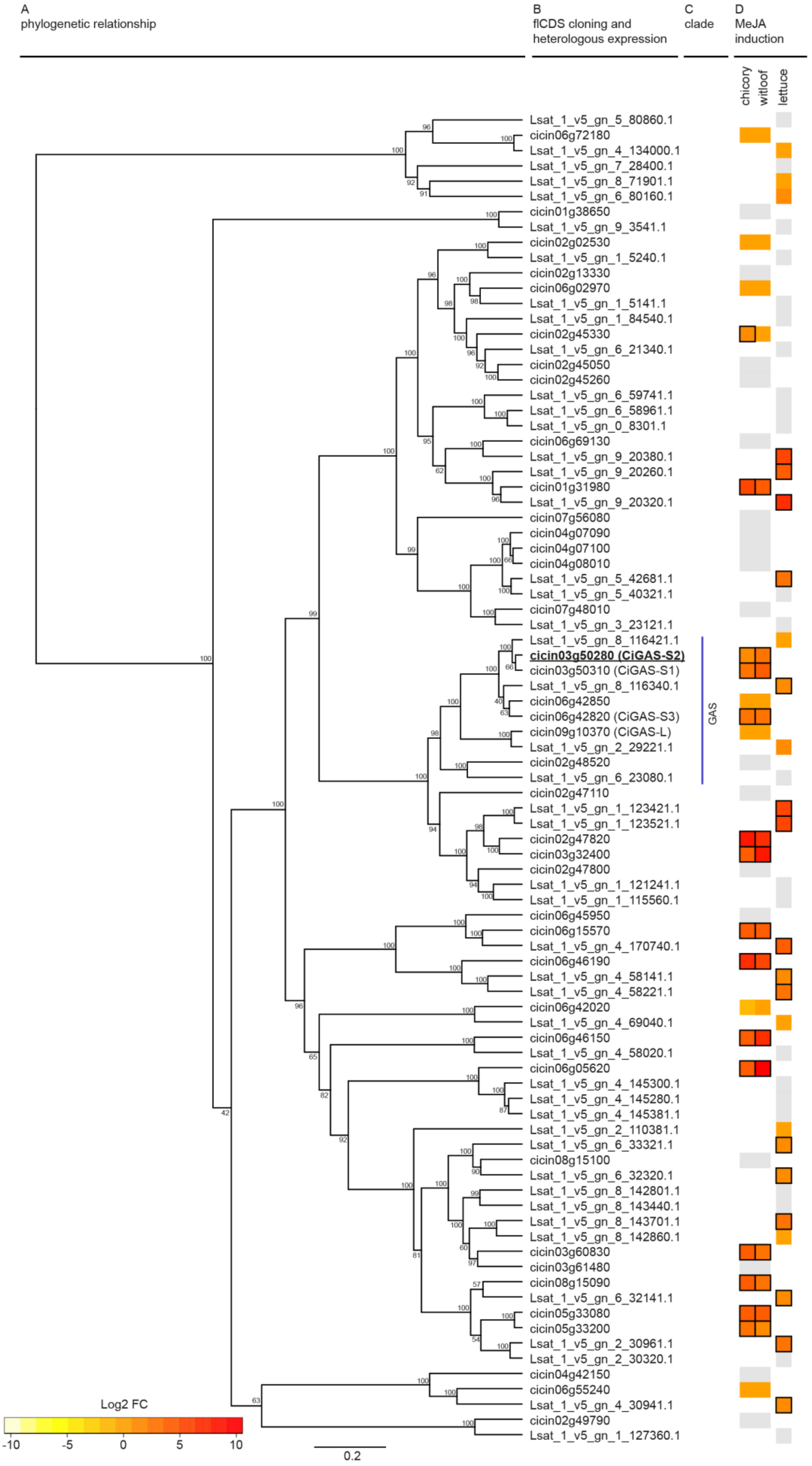

A
phylogenetic relationship
B
flCDS cloning and heterologous expression
C
clade
D
MeJA induction
chicory
witloof
lettuce
Lsat_1_v5_gn_5_80860.1
cicin06g72180
Lsat_1_v5_gn_4_134000.1
Lsat_1_v5_gn_7_28400.1
Lsat_1_v5_gn_8_71901.1
Lsat_1_v5_gn_6_80160.1
cicin01g38650
Lsat_1_v5_gn_9_3541.1
cicin02g02530
Lsat_1_v5_gn_1_5240.1
cicin02g13330
cicin06g02970
Lsat_1_v5_gn_1_5141.1
Lsat_1_v5_gn_1_84540.1
cicin02g45330
Lsat_1_v5_gn_6_21340.1
cicin02g45050
cicin02g45260
Lsat_1_v5_gn_6_59741.1
Lsat_1_v5_gn_6_58961.1
Lsat_1_v5_gn_0_8301.1
cicin06g69130
Lsat_1_v5_gn_9_20380.1
Lsat_1_v5_gn_9_20260.1
cicin01g31980
Lsat_1_v5_gn_9_20320.1
cicin07g56080
cicin04g07090
cicin04g07100
cicin04g08010
Lsat_1_v5_gn_5_42681.1
Lsat_1_v5_gn_5_40321.1
cicin07g48010
Lsat_1_v5_gn_3_23121.1
Lsat_1_v5_gn_8_116421.1
cicin03g50280 (CiGAS-S2)
cicin03g50310 (CiGAS-S1)
Lsat_1_v5_gn_8_116340.1
cicin06g42850
cicin06g42820 (CiGAS-S3)
cicin09g10370 (CiGAS-L)
Lsat_1_v5_gn_2_29221.1
cicin02g48520
Lsat_1_v5_gn_6_23080.1
GAS
cicin02g47110
Lsat_1_v5_gn_1_123421.1
Lsat_1_v5_gn_1_123521.1
cicin02g47820
cicin03g32400
cicin02g47800
Lsat_1_v5_gn_1_121241.1
Lsat_1_v5_gn_1_115560.1
cicin06g45950
cicin06g15570
Lsat_1_v5_gn_4_170740.1
cicin06g46190
Lsat_1_v5_gn_4_58141.1
Lsat_1_v5_gn_4_58221.1
cicin06g42020
Lsat_1_v5_gn_4_69040.1
cicin06g46150
Lsat_1_v5_gn_4_58020.1
cicin06g05620
Lsat_1_v5_gn_4_145300.1
Lsat_1_v5_gn_4_145280.1
Lsat_1_v5_gn_4_145381.1
Lsat_1_v5_gn_2_110381.1
Lsat_1_v5_gn_6_33321.1
cicin08g15100
Lsat_1_v5_gn_6_32320.1
Lsat_1_v5_gn_8_142801.1
Lsat_1_v5_gn_8_143440.1
Lsat_1_v5_gn_8_143701.1
Lsat_1_v5_gn_8_142860.1
cicin03g60830
cicin03g61480
cicin08g15090
Lsat_1_v5_gn_6_32141.1
cicin05g33080
cicin05g33200
Lsat_1_v5_gn_2_30961.1
Lsat_1_v5_gn_2_30320.1
cicin04g42150
cicin06g55240
Lsat_1_v5_gn_4_30941.1
cicin02g49790
Lsat_1_v5_gn_1_127360.1
Log2 FC
-10
-5
0
5
10
0.2

Supplementary Figure 2. Phylogenetic analysis of *C. intybus* and *L. sativa* terpene synthase genes (*TPS*s) together with MeJA induction of gene expression in industrial chicory, witloof and lettuce. (A) The protein sequences were aligned using MUSCLE with default parameters and the tree was inferred with the UPGMA method. Bootstrap values (shown at the branching points) are based on 1,000 replicates. (B) *Cicin03g50280* and *cicin09g10370* correspond to the previously identified functional *CiGASs* (AAM21659.1) (later referred to as *CiGAS-S2* by Cankar et al. (2021)) and *CiGASl* (AAM21658.1) (later referred to as *CiGAS-L* by Cankar et al. (2021)), respectively, while *cicin03g50310* corresponds to *CiGAS-S1*, and *cicin06g42820* corresponds to *CiGAS-S3* (Cankar et al., 2021)). The full-length CDS (flCDS) of *CiGASs* (bold and underlined) was synthesized and used for heterologous expression by co-agroinfiltration in *N. benthamiana*. (C) The candidate GAS clade is indicated by the blue vertical bar. (D) Heatmap of gene expression response to MeJA treatment (log2-fold change in gene expression versus mock treatment as measured by RNA-seq). Gray boxes indicate non-expressed genes, while MeJA-inducible genes with log2 fold change ≥ 1 are marked with a black outline.

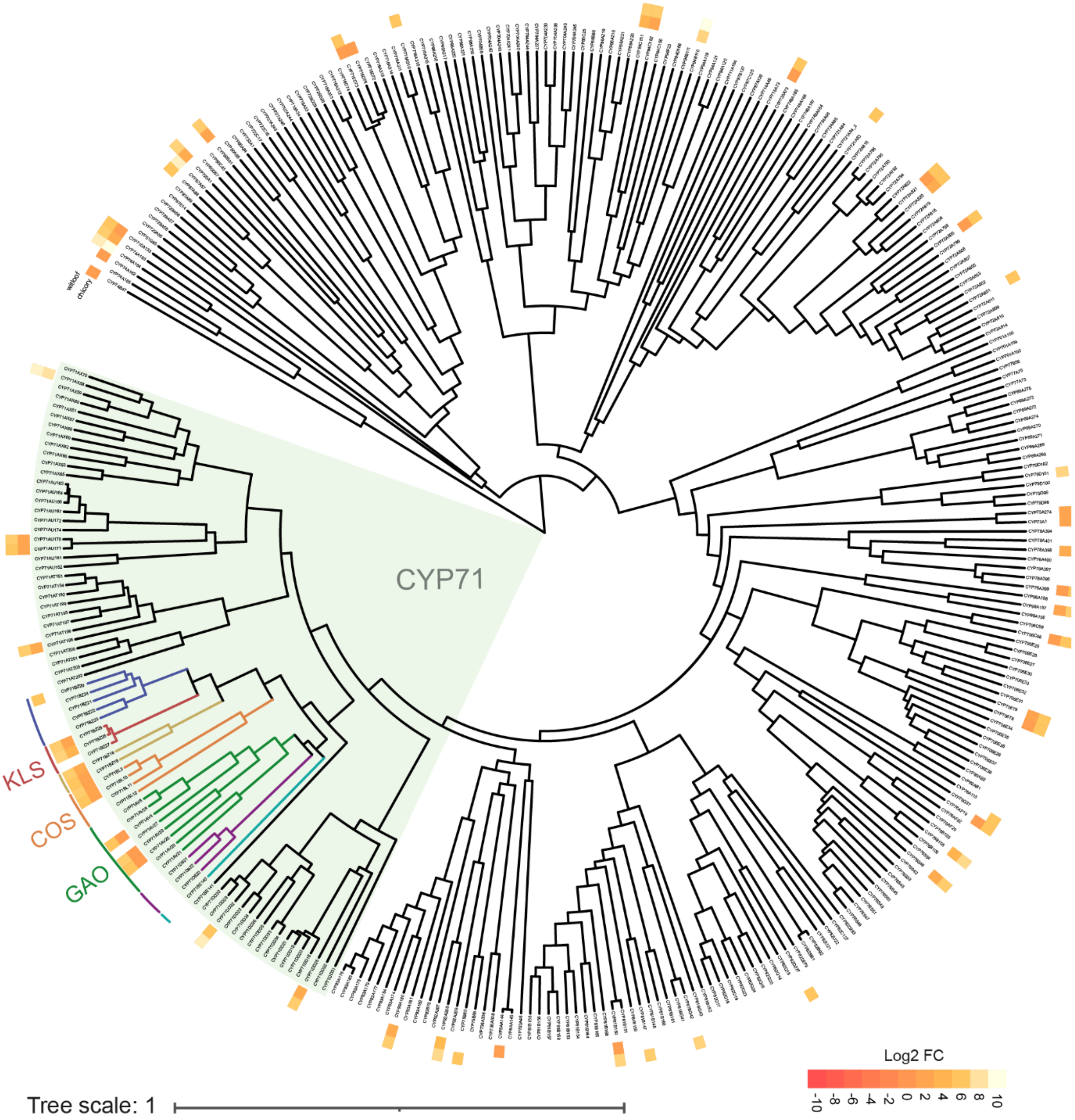

Supplementary Figure 3. Phylogenetic analysis of the 320 cytochrome P450 (*CYP*) *C. intybus* genes (excluding pseudogenes). The protein sequences were aligned using MUSCLE with default parameters and the tree was inferred with the UPGMA method. Tree decorations show the heatmap with significant response to MeJA (log2-fold change in gene expression after MeJA versus mock treatment > 1) in industrial chicory and witloof and were added in iTOL (https://itol.embl.de). The green slice indicates the *CYP71* gene family member clade. *CYP71AV4*/*CYP71AV8* and *CYP71BL3* correspond to the previously identified functional *CiGAO* (ADF43080.1) and *CiCOS* (AEG79727.1), respectively. Also *CiKLS1*, *CiKLS2* and *CiKLS3* are previously described as functional.

A

I/S	cicin02g48520	cicin09g10370	CiGASl	cicin03g50310	cicin03g50280	CiGASs	cicin06g42850	cicin06g42820	cicin02g47110	cicin02g47800	cicin02g47820	cicin03g32400
cicin02g48520	—	85.7	85.7	88.0	87.9	87.8	84.1	87.7	84.1	83.1	82.9	82.6
cicin09g10370	60.3	—	100.0	89.0	88.6	88.6	84.1	88.6	82.8	81.8	81.3	79.8
CiGASl	60.3	99.7	—	88.8	88.5	88.4	83.9	88.5	82.8	81.8	81.1	79.6
cicin03g50310	65.0	69.9	69.7	—	99.6	99.6	93.0	98.4	88.2	86.2	86.6	84.7
cicin03g50280	65.1	70.1	69.9	98.9	—	100.0	92.8	98.2	88.0	85.8	86.5	84.5
CiGASs	65.1	70.2	70.0	98.9	100.0	—	92.8	98.2	88.0	85.8	86.4	84.5
cicin06g42850	60.0	65.1	64.9	83.8	83.6	83.6	—	94.2	83.8	81.4	82.7	81.2
cicin06g42820	64.4	70.2	70.1	91.8	91.8	91.8	90.2	—	87.8	85.5	86.5	84.8
cicin02g47110	59.3	55.7	55.5	62.1	62.1	62.1	57.7	62.1	—	84.8	85.9	83.9
cicin02g47800	59.0	54.5	54.3	64.4	64.2	64.4	59.4	62.8	62.9	—	89.8	88.2
cicin02g47820	58.8	53.7	53.5	63.6	63.5	63.6	58.3	61.5	64.3	75.5	—	95.9
cicin03g32400	58.8	52.5	52.3	61.1	61.1	61.3	56.3	60.2	61.8	73.2	88.3	—

B

I/S	CYP71AV20	CYP71AV21	CYP71AV27	CiGAO	CYP71AV4	CYP71AV8	CYP71AV26	CYP71AV23	CYP71AV25
CYP71AV20	—	91.0	79.8	82.1	81.9	83.7	83.1	81.8	82.0
CYP71AV21	71.7	—	81.2	81.1	80.9	83.7	83.1	81.6	81.8
CYP71AV27	51.7	52.5	—	86.8	86.8	86.9	86.5	83.6	83.4
CiGAO	53.4	54.0	61.8	—	100.0	95.7	96.3	90.0	90.4
CYP71AV4	53.4	54.2	61.8	99.6	—	95.7	96.3	90.0	90.2
CYP71AV8	56.0	55.4	63.1	82.2	82.4	—	98.4	91.2	90.8
CYP71AV26	55.4	55.4	63.7	82.6	82.8	95.4	—	90.8	90.6
CYP71AV23	49.9	50.9	51.5	61.9	62.3	62.7	62.2	—	93.0
CYP71AV25	51.7	52.3	55.6	66.0	66.2	66.3	66.7	77.9	—

C

I \ S	CYP71BL12	CYP71BL11	CYP71BL10	CYP71BL3	CiCOS
CYP71BL12		83.5	84.1	84.5	84.7
CYP71BL11	57.2		98.0	97.8	97.8
CYP71BL10	56.0	90.7		98.8	98.8
CYP71BL3	55.5	89.5	95.8		100.0
CiCOS	55.4	89.7	96.0	99.8	

D

I \ S	CYP71 BZ18	CYP71 BZ19	TpKLS	CYP71 BZ27	CYP71 BZ25	CYP71 BZ26	CYP71 BZ21	CYP71 BZ22	CYP71 BZ23	CYP71 BZ20	CYP71 BZ24
CYP71 BZ18		94.4	84.4	83.2	83.4	83.0	87.5	88.8	87.8	88.0	88.2
CYP71 BZ19	78.6		85.0	84.6	85.0	84.8	88.8	89.0	87.8	88.6	90.0
TpKLS	52.9	54.3		88.0	88.2	88.2	85.7	88.2	86.4	87.4	87.4
CYP71 BZ27	55.1	55.5	61.6		100.0	100.0	85.7	88.2	86.2	88.8	88.2
CYP71 BZ25	55.3	55.9	62.0	98.6		100.0	86.1	88.4	86.4	89.0	88.2
CYP71 BZ26	55.5	55.7	61.8	98.4	99.0		86.1	88.4	86.4	89.0	88.2
CYP71 BZ21	61.0	62.4	61.6	65.7	65.9	66.3		94.8	92.8	94.0	94.4
CYP71 BZ22	62.7	64.3	63.1	65.9	65.9	66.1	82.1		96.4	96.4	95.4
CYP71 BZ23	61.8	62.6	62.2	62.8	62.8	62.8	77.7	90.2		94.0	93.8
CYP71 BZ20	60.5	61.5	61.9	66.1	66.1	66.3	83.1	85.2	80.6		96.0
CYP71 BZ24	61.7	63.1	63.9	65.9	65.9	66.3	83.7	86.2	80.6	86.4	

Supplementary Figure 4. % identity matrix (I) and % similarity matrix (S) (with Blosum62) of *C. intybus* paralogous proteins in a protein alignment containing **(A)** CiGASl and CiGASs, **(B)** CiGAO, **(C)** CiCOS and **(D)** TpKLS and CYP71BZ.

Supplementary Table 1. Primers of the (candidate) reference genes and *GASs*, *GASl*, *GAO* and *COS* genes of *C. intybus* and *L. sativa* for RT-qPCR analysis.

Species		Gene	GenBank nr.	Study	Forward primer	Reverse primer
C. intybus	1	*ACT2*	KP752080	(Delporte et al., 2015)	5'-AGGATCTTCAGCCCCTTGTT-3'	5'-ACCATTGTCTGGCAGCCTAC-3'
	2	*UBQ10*	KP752078	(Delporte et al., 2015)	5'-GCGTTTGTTCATTGCTTCAA-3'	5'-ATGGTGTCCGAGATTTCCAC-3'
	3	*TIP41*	EH681285.1	(Delporte et al., 2015)	5'-GTTGGGTGCGCATCTCTAAT-3'	5'-AGCTCCGGCAGCTTTTACTT-3'
	4	*UBC*	EH709287.1	(Delporte et al., 2015)	5'-GGTGGAGTTTTTCAGCTTGC-3'	5'-TCAAGGCAAATCTCTCCTGTC-3'
	5	*PP2AA3*	EH675923.1	(Delporte et al., 2015)	5'-TGCTTACCCTAGTGCCTCTGA-3'	5'-TTCCCAAATTTGTAGCAGCA-3'
	6	*CYP5*	EH688850.1	(Delporte et al., 2015)	5'-CAGTGCCAAAAACAGCAGAA-3'	5'-GGTGAAGTCACCTCCCTGAA-3'
	7	*SAND*	KP752083	(Delporte et al., 2015)	5'-TGCTTACACCACAAGGCAAG-3'	5'-GAAGCAGCATGTCATCAGGA-3'
	8	*Clath*	EH684142.1	(Delporte et al., 2015)	5'-TGCTTCCGCCATCTACTTTT-3'	5'-TCCCAAGTTCCTTTGTTTGC-3'
	9	*PP2AA2*	FL675704.1	(Delporte et al., 2015)	5'-CATGGGCTCAGAAATCACCT-3'	5'-ATTGGTCAACGATGGGGATA-3'
	10	*bTUB*	KP752084	(Delporte et al., 2015)	5'-TTTCCCGGTCAACTCAACTC-3'	5'-ACTGTGAGGGCTCGGTATTG-3'
	11	*PROF*	DT2111339.1	(Delporte et al., 2015)	5'-CCCAATTTCCCTCAGTTGAA-3'	5'-TTGGTCCCACCACAGTGTAA-3'
	12	*CiGASs*	AF497999.1	(This study)	5'-CCTCCTTCTGTATGGGGTGA-3	5'-TTCTTCTTTTGGCTCCTCCA-3
	13	*CiGASl*	AF498000.1	(This study)	5'-GCTTTAGCATTCACCGAAGC-3	5'-GTGCCTCTACCATTGGCATT-3
	14	*CiGAO*	GU256644.1	(This study)	5'-GTGTCGACAATCGTGGTGTC-3	5'-CCGTAGGGTGCAAGAACAAT-3
	15	*CiCOS*	JF816041.1	(This study)	5'-TCGGTGGTGAAGGGTAAGAC-3	5'-AGGTGTCGGAGCATGTAACC-3
L. sativa	1	*APT1*	AT1G27450	(Borowski et al., 2014)	5'-CTGTACAAGAAGGAGAACGAGC-3'	5'-ACGAGCACATACAGTGGCTT-3'
	2	*EIF2A*	EU028334	(Borowski et al., 2014)	5'-TAGGCGAGTGGAGAAGCATT-3'	5'-GTAGAAACAGCAACAGGCAAA-3'
	3	*TIP41*	CST1.6123	(Borowski et al., 2014)	5'-GAGAGATTTGCTGGAGGGAAACTA-3'	5'-CCTTTGACTGATGATGTTTGGA-3'
	4	*UBQ*	GW397659	(Borowski et al., 2014)	5'-AAGACCTACACCAAGCCCAA-3'	5'-AAGTGAGCCCACACTTACGA-3'
	5	*ACT2*	AK317453.1	(Sgamma et al., 2016)	5'-CAAGGGCAGTGTTTCCTAGTATTG-3'	5'-CCTCATCTCCAACATAAGCATCTTTC-3'
	6	*TUA-3*	BT000718.1	(Sgamma et al., 2016)	5'-CTTCTTAGTGTTCAATGCTGTTGG-3'	5'-GAAGGGTAGATAGTGAAACCGAGC-3'
	7	*GAPDH*	AK317337.1	(Sgamma et al., 2016)	5'-TTAAAGGGTGGTGCTAAGAAGGTCAT-3'	5'-AGCTCAGGTTTGTATTCGTTCTCATT-3'
	8	*UBC9*	AF325019.2	(Sgamma et al., 2016)	5'-AAGGTATTGTTGTCGATTTGCTCTCT-3'	5'-TCCTTGCAGTTGTCTCATACTTGTTC-3'
	9	*PP2AA3*	BT002601.1	(Sgamma et al., 2016)	5'-CATGCAATGGTTACAAGACAAGGTAT-3'	5'-CAAACTCCTCCGCAAGTCTCTTC-3'
	10	*PP2A-1*	AY096543.1	(Sgamma et al., 2016)	5'-ATTCATGGTCAATTCTACGATCTGGT-3'	5'-GAATAATACCCGCGATCAACATAATC-3'

Species		Gene	GenBank nr.	Study	Forward primer	Reverse primer
	11	*LsGASs*	LOC111882428	(This study)	5'-TCCGGAATACATTCGACCAT-3	5'-TGAAGCAATGACGCTTTTTG-3
	12	*LsGASl*	LOC111909135	(This study)	5'-CAAGCCATGAGTCGGTTGTA-3	5'-TTTCCACCACTGTGAAACCA-3
	13	*LsGAO*	LOC111882413	(This study)	5'-AGCAAAGCCAACGAGACACT-3	5'-TTCCAGCTCCGAACATATCC-3
	14	*LsCOS*	LOC111890503	(This study)	5'-TTGTGGAACAGACCCTGACA-3	5'-TCGAAATCTGCACCCATGTA-3

Supplementary Table 2 Gene-specific primers for the amplification of full-length coding sequences in *C. intybus* using Q5 High Fidelity Polymerase.

Primer name	Sequence without AttB sites	Gene clade
CYP71BZ18_F	5'-ATGGACACACAAATAGCATT-3'	*BZ-I*
CYP71BZ18_R	5'-TTAATTGTCGGGAGCATAAGTTG-3'	*BZ-I*
CYP71BZ19_F	5'-ATGAGCACCGAATTAGCATTCTC-3'	*BZ-I*
CYP71BZ19_R	5'-TCAACTGTAAGAAGCATAAGAAG-3'	*BZ-I*
CYP71BZ20_F	5'-ATGGACACTGATATCACCTTCT-3'	*BZ-II*
CYP71BZ20_R	5'-TCATTGTTTTATTGTCAAAA-3'	*BZ-II*
CYP71BZ21_F	5'-ATGGACGCCGTCGCCGGTAT-3'	*BZ-II*
CYP71BZ21_R	5'-TTAAAATGAAGAATATATAGTTG-3'	*BZ-II*
CYP71BZ22_CYP71BZ24_F	5'-ATGGACGCCGATATCACCTTC-3'	*BZ-II*
CYP71BZ22_R	5'-TCAAACTGAAAAATAGAGAGTTG-3'	*BZ-II*
CYP71BZ23_F	5'-ATGGACCTCGCTATCACCTAC-3'	*BZ-II*
CYP71BZ23_R	5'-TCAAGACGAAGAATAGAGTGTCG-3'	*BZ-II*
CYP71BZ24_R	5'-TCAAAATGAAGAATATAGAGTTG-3'	*BZ-II*
CYP71BZ25_F	5'-ATGGCCATCGAAATGACCATC-3'	*CiKLS*
CYP71BZ25_R	5'-TCAAAATGGAATAAAAACGGT-3'	*CiKLS*
CYP71BZ26_F	5'-ATGGTCATCGATATGACCATCGT-3'	*CiKLS*
CYP71BZ26_R	5'-TCAAAACGGGATAAAAATTGTTG-3'	*CiKLS*
CYP71BZ27_F	5'-ATGGCCATCGATATGACCATCG-3'	*CiKLS*
CYP71BZ27_R	5'-TCAAAACGGGATAAAAACTGTTG-3'	*CiKLS*
CYP71BL3_F	5'-ATGGAGCCTCTCACCATCGT-3'	*CiCOS*
CYP71BL3_R	5'-TTACTTTGCTATGGACTTGGGGA-3'	*CiCOS*
CYP71BL10_F	5'-ATGGAGCCTTTCACCATCGT-3'	*CiCOS*
CYP71BL10_R	5'-CTACTTCCCTAACGACTTGGG-3'	*CiCOS*
CYP71BL11_F	5'-ATGGACCCTCTCACTATCTTCTC-3'	*CiCOS*
CYP71BL11_R	5'-CTAGCTTGATAACGACCTTGGGA-3'	*CiCOS*
CYP71BL12_F	5'-ATGGATTCCATAGACACCTTCTC-3'	*CiCOS*
CYP71BL12_R	5'-TTAGTTCTGTAGTGCTGAATGAG-3'	*CiCOS*
CYP71AV25_F	5'-ATGGAACTCTCTCTCCTCAT-3'	*CiGAO*
CYP71AV25_R	5'-TTAGAAGATTGGCACTAAAAACA-3'	*CiGAO*
CYP71AV26_F	5'-ATGGAGGTTTCTGTAACCGCT-3'	*CiGAO*
CYP71AV26_R	5'-TTAAATAGATTCTTCAAGTGTTT-3'	*CiGAO*
CYP71AV8_F	5'-ATGGAGATTTCTATCCCCACT-3'	*CiGAO*
CYP71AV8_R	5'-TCAAGTAGATGCCGTAAGTGT-3'	*CiGAO*
CYP71AV27_F	5'-ATGGAATTCTCTCTCGCTGGC-3'	*CiGAO*
CYP71AV27_R	5'-CTAGTTTTTCGCGAAGTTGTGTG-3'	*CiGAO*
CYP71AV23_F	5'-ATGGAACTCTCTCTCGTCACT-3'	*CiGAO*
CYP71AV23_R	5'-TTAGAAGGTTGGTACTAAAAACA-3'	*CiGAO*
CYP71AV4_F	5'-ATGGAGCTCTCACTCACTACT-3'	*CiGAO*
CYP71AV4_R	5'-TTAAAAACTTGGTACGAGTATCA-3'	*CiGAO*

Supplementary Table 3 Primers used for the generation of the gene-specific gRNAs.

Gene clade	Multiplex CRISPR group	Target gene locus	Locus	gRNA_	Sequence
AV (CiGAO)	AV_A	CYP71AV26_CYP71AV8	locus 1	gRNA_39	AGAACCTCTTTTGCCCAACG
		CYP71AV27	locus 1	gRNA_41	AAGACCTCTTTCGCCCAGCG
		CYP71AV4	locus 1	gRNA_43	AAAATCTCTTTAGCCCATTT
	AV_B	CYP71AV23_CYP71AV25	locus 1	gRNA_37	GGAGTGTTGGAATTTTGTTC
BL (CiCOS)	BL	CYP71BL3_CYP71BL10_CYP71BL12	locus 1	gRNA_29	AGCTGTAGGTGCATGATGGG
		CYP71BL11	locus 1	gRNA_31	CCCATCATGCACTTGAAACT
		CYP71BL3_CYP71BL10_CYP71BL12	locus 2	gRNA_33	TTTCTGCATCTTCAACCACT
		CYP71BL11	locus 2	gRNA_35	TGTTGGGGATAAAATCTAAG
BZ (I) (CiKLS)	BZ-I	CYP71BZ25_CYP71BZ26_CYP71BZ27	locus 1	gRNA_17	AAGTGGTGGCATTATCGAGT
		CYP71BZ25_CYP71BZ26_CYP71BZ27	locus 1	gRNA_19	GGAAACTTAACTTAGGAAAC
BZ (II)	BZ-II	CYP71BZ21_CYP71BZ22_CYP71BZ23_CYP71BZ24	locus 1	gRNA_21	GTAGTTTTGGTGGGCCTGGT
		CYP71BZ22_CYP71BZ23_CYP71BZ24	locus 2	gRNA_25	ATCGTGTGCAAATCTTCGTT
		CYP71BZ20	locus 1	gRNA_23	GTAGTTTTGGTGGCCCTGGT
		CYP71BZ20_CYP71BZ21	locus 2	gRNA_27	ATCGTGTGCAAATCGTCGTT

Supplementary Table 4 Primers used for HiPlex amplicon sequencing of the CRISPR/Cas mutants.

Gene clade	Multiplex CRISPR group	Target gene locus	Locus	Primer name	Sequence
AV (CiGAO)	AV	CYP71AV23_F	locus 1	primer_055_F	AGCTTTGCACCTTGGAACTTTT
		CYP71AV25_F	locus 1	primer_057_F	AGCTTTGCACCTTGGAGCTTTT
		CYP71AV26_F	locus 1	primer_061_F	GCCAGGAAGTACGGATCTCTC
		CYP71AV8_F	locus 1	primer_062_F	GCCAGGAAGCATGGATCTCT
		CYP71AV27_F	locus 1	primer_063_F	GCCAGGAAATATGGACCTCT
		CYP71AV4_F	locus 1	primer_064_F	GCCAGAAAGCATGGATCTTTGA
		CYP71AV23_R	locus 1	primer_058_R	TGGATGTTGTAAATGTAGTGGGA
		CYP71AV25_R	locus 1	primer_060_R	GTCCTGCTGTAAATGCAGTGGGA
		CYP71AV26_R	locus 1	primer_065_R	CACCGGTTAAAGTCTCGGGT
		CYP71AV8_R	locus 1	primer_066_R	CGGTTAAAGTCTCCGGTCTGT
		CYP71AV27_R	locus 1	primer_067_R	TGGTTAGAACCACAGGTCTGT
		CYP71AV4_R	locus 1	primer_068_R	CCGGTTAGGGTCTCAGGTCT
BL (CiCOS)	BL	CYP71BL3_CYP71BL11_F	locus 1	primer_001_F	GGGCCACCAAAACTACCCAT
		CYP71BL10_F	locus 1	primer_002_F	GGGCCACCGAAATTACCCAT
		CYP71BL12_F	locus 1	primer_003_F	GGGCCTCAAACCCTACCTCT
		CYP71BL3_F	locus 2	primer_011_F	CTGGCATCGTATTATCCGGGT
		CYP71BL10_F	locus 2	primer_012_F	CTGGCATCGTATTATCCTCGT
		CYP71BL11_F	locus 2	primer_013_F	CTGGCGTCATATTATCCTGGT
		CYP71BL12_F	locus 2	primer_014_F	GGGTGAATTCTTTCCTCGGC
		CYP71BL3_CYP71BL10_R	locus 1	primer_004_R	TCGAGGTGTAGAGACGACGA
		CYP71BL11_R	locus 1	primer_005_R	TCGTGGTGTAGAGACGACGA
		CYP71BL12_R	locus 1	primer_006_R	TCGAGGTGATGAAACCACGA
		CYP71BL3_CYP71BL10_R	locus 2	primer_015_R	TCTTGATCGCTCTTGTTACTTCCT
		CYP71BL11_R	locus 2	primer_016_R	TCTTGGTCATTGTTCTTTCTTCCT
		CYP71BL12_R	locus 2	primer_017_R	CGGCTCTCTCAATCTCACCA
BZ (I) (CiKLS)	BZ-I	CYP71BZ25_ CYP71BZ26_ CYP71BZ27_F	locus 1	Primer_069_F	TGCAGACAACAAGACGTATTGA
		CYP71BZ25_ CYP71BZ26_ CYP71BZ27_R	locus 1	Primer_070_R	AGTGTCTCGTGAATATGCGTC

Gene clade	Multiplex CRISPR group	Target gene locus	Locus	Primer name	Sequence
BZ (II)	BZ-II	CYP71BZ22_CYP71BZ23_F	locus 1	primer_030_F	CCTCTCTCATTCTTTCGTTCT
		CYP71BZ24_F	locus 1	primer_031_F	CCTCTCTCATATTTTCATTCT
		CYP71BZ20_F	locus 1	primer_032_F	CATCTCTCATTCTTTCCTTCT
		CYP71BZ21_F	locus 1	primer_033_F	CCTCTCTCATTTTCTCATTCT
		CYP71BZ22_CYP71BZ23_F	locus 2	primer_048_F	GACCTGTGGAAAGCCGGTAA
		CYP71BZ24_F	locus 2	primer_049_F	GTCCTGTGGAAAGCCGGTAA
		CYP71BZ20_F	locus 2	primer_050_F	GAACTGTGGAAAGCCGGTGA
		CYP71BZ21_F	locus 2	primer_051_F	CAACTGTGGAAAGCCGGTAA
		CYP71BZ22_R	locus 1	primer_034_R	AATCACGGAATGCATGGTGC
		CYP71BZ23_R	locus 1	primer_035_R	AATCACGTAATGCATGGTGC
		CYP71BZ20_CYP71BZ24_R	locus 1	primer_036_R	ATTCACGGAATGCATGGTGC
		CYP71BZ21_R	locus 1	primer_037_R	AGTCACGGAATGCATGGTGC
		CYP71BZ22_CYP71BZ23_CYP71BZ24_R	locus 2	primer_052_R	ATTTCCCCAACTCGTCCACC
		CYP71BZ20_R	locus 2	primer_053_R	ATCTTCCCAACTCATCGATC
		CYP71BZ21_R	locus 2	primer_054_R	ATCTCCCTATTTCGTCCACC
		CYP71BZ22_R	locus 1	primer_034_R	AATCACGGAATGCATGGTGC

Supplementary Table 5. Overview and number of *CiGAO*, *CiCOS* and *CiKLS* mutated plant genotypes, separated in groups shown in **Supplementary Figure 1**. LOF type numbers refer to the L-numbers in Figure 2.

Targeting gene clade	LOF type	Mutation type	Plant genotype					# Plants (# metabolite profiled plants)
			**CYP71AV4**	**CYP71AV8**	**CYP71AV27**	**CYP71AV23**	**CYP71AV25**	
*CiGAO*_A	L26	M1	-	-	D13 / I1			2
	L25	M2	-	-	D12 / I1			3
*CiGAO*_B	L27	M3				-	D13 / WT	9 (1)
*CiGAO*_A&B	L28	M4	I1 / WT	-	-	-	-	5 (1)
	L29	M5	-	D8 / WT	I1	D12 / WT	D27 / WT	2 (1)
			**CYP71BZ25**	**CYP71BZ26**	**CYP71BZ27**			
CiKLS	L4	M6	D10D8	D9I1T	D31 / D34			5 (2)
	L4	M7	D26 / D8	D30 / D41I11	D26 / D47			3
	L4	M8	D43I25	D9I1	D31 / D34			2
	L3	M9	WT / D31	WT / D20	WT / I1 / D11			7
	L3	M10	WT / D31	WT / D9I1	WT / D31 / D34			1
	L2	M44	-	WT / mix	-			5
	L1	M45	-	-	WT / D31 / D34			2
			**CYP71BZ20**	**CYP71BZ21**	**CYP71BZ22**	**CYP71BZ23**	**CYP71BZ24**	
BZ-II_A	L10	M11			D8	D2	D1 / D5	2 (1)

	L10	M12			D8	D2 / D10	D1 / D5	8 (3)
	L5	M13			WT / I1	WT / D8	-	1 (1)
	L8	M14			D8 / D10	D6 / I1	WT / D4	5 (3)
	L7	M15			WT / D8	WT/D2/D10	WT/D1/D5	4
	L9	M16			WT / D8	D2 / D10	D1 / D5	1 (1)
	L7	M46			WT / D10 / D8	WT / D6 / I1	WT / D4	2 (1)
BZ-II_B	L12	M17	D54I8 // D9	-				4
	L14	M18	D3 / D7	D12				1
	L11	M20	WT / D1	-				18 (3)
	L13	M21	-	D9				4
	L15	M22	D6 / D1	WT / D1				1 (1)
	L11	M24	WT / D1 // WT / I1	-				5 (1)
	L11	M25	D5I1/WT	-				1 (1)
BZ-II_A&B	L18	M19	D7 / WT	-	-	D10 / WT	D1 / WT	3
	L19	M26	D5I1 // D6 / WT	-	D4	D10	D12	25
	L19	M27	D5I1	-	D4	D10	D12	6 (1)
	L17	M28	D5I1	-	-	-	I1 / D14	4
	L16	M29	-	WT / I1	-	-	D2I1 / WT	1
	L16	M30	-	WT / I1	-	-	WT / I1	3 (1)
	L6	M31	-	-	WT / D1	D5 / D14 // WT / I1A	-	2
	L20	M32	-	WT / D4	D9 // D3 / WT	D2 / I6	D2	2
	L21	M33	D12I52	D4 / WT	D12 // D3 / WT	D4 / D12	D13	2 (1)

			CYP71BL3	CYP71BL12	CYP71BL10	CYP71BL11	
CiCOS	L22	M34	-	WT/D7	-	-	31 (2)
	L23	M35	-	D11/D16I25 // D7	-	-	4
	L23	M47	-	I1D15 // D11	-	-	2
	L23	M36	-	D7	-	-	3
	L23	M37	-	D4	-	-	4
	L23	M38	-	D5	-	-	5 (3)
	L23	M39	-	D8 // D16	-	-	19 (5)
	L23	M40	-	D16	-	-	1 (1)
	L23	M41	-	D8 // D5	-	-	1
	L23	M42	-	D1I2 // D1	-	-	1 (1)
	L24	M43	D13/WT	I1/D1I2 // D4	-		4 (1)

WT / Indel = Heterozygous mutation; Indel = Single observed mutation; Indel / Indel = Compound heterozygous mutation; I = Insertion; D = Deletion, WT = Wild Type, - = only WT alleles; '//' separates mutation data of two analyzed loci (see Supplementary Figure 4). Mutation types and plant genotypes with mutations leading to early stop codons, truncating the translated protein, are underlined. '-' indicates no detected mutation. Numbers between brackets indicate the number of plant lines that were metabolite profiled.

Supplementary Table 6. Overview of number of annotated genes, expressed genes and differentially expressed genes (DEGs) (p_{adj}<0.05) between MeJA and mock treatment, MeJA downregulated and upregulated DEGs, and MeJA upregulated DEGs belonging to the *TPS* and *CYP* families (excluding pseudogenes), with the total number of *TPS* and *CYP* genes between brackets, in industrial chicory, witloof and lettuce. RNA-seq reads from industrial chicory and witloof were mapped to the CDS of *C. intybus*, while lettuce RNA-seq reads were mapped to the CDS of *L. sativa*. Upregulated genes have a log2 fold change ≥ 1, while downregulated genes have a log2 fold change ≤ -1. The number of annotated genes of industrial chicory and witloof are equal because the same CDS gene set was used from the *C. intybus* var. *sativum* reference genome.

	**Industrial chicory**	**Witloof**	**Lettuce**
**# Genes**	53,507	53,507	37,829
**# Expressed genes**	20,358	20,024	18,313
**# DEGs**	3,384	2,696	1,395
**# Downregulated DEGs**	1,489	1,076	586
**# Upregulated DEGs**	1,895	1,620	809
**# Upregulated TPS DEGs**	15 (40)	14 (40)	16 (47)
**# Upregulated CYP DEGs**	49 (320)	51 (320)	33 (354)

Supplementary Table 7. Overview of *N. benthamiana* expression assays for the validation of paralogous *CiGAO* and *CiCOS* genes.

Assay	Co-infiltrated *N. benthamiana* expression vectors
Empty vector	pEAQ-HT-DEST1[tHMGR1]; pEAQ-HT-DEST1[Empty]
CiCOS	pEAQ-HT-DEST1[tHMGR1]; pEAQ-HT-DEST1[CiGASs]; pEAQ-HT-DEST1[CYP71AV4]; pEAQ-HT-DEST1[CYP71BL3]
CYP71AV27	pEAQ-HT-DEST1[tHMGR1]; pEAQ-HT-DEST1[CiGASs]; pEAQ-HT-DEST1[CYP71AV27]; pEAQ-HT-DEST1[CYP71BL3]
CYP71AV8	pEAQ-HT-DEST1[tHMGR1]; pEAQ-HT-DEST1[CiGASs]; pEAQ-HT-DEST1[CYP71AV8]; pEAQ-HT-DEST1[CYP71BL3]
CYP71AV26	pEAQ-HT-DEST1[tHMGR1]; pEAQ-HT-DEST1[CiGASs]; pEAQ-HT-DEST1[CYP71AV26]; pEAQ-HT-DEST1[CYP71BL3]
CYP71AV25	pEAQ-HT-DEST1 [tHMGR1]; pEAQ-HT-DEST1[CiGASs]; pEAQ-HT-DEST1[CYP71AV25]; pEAQ-HT-DEST1[CYP71BL3]
CYP71BL10	pEAQ-HT-DEST1tHMGR1]; pEAQ-HT-DEST1[CiGASs]; pEAQ-HT-DEST1[CYP71AV4]; pEAQ-HT-DEST1[CYP71BL10]
CYP71BL11	pEAQ-HT-DEST1tHMGR1]; pEAQ-HT-DEST1[CiGASs]; pEAQ-HT-DEST1[CYP71AV4]; pEAQ-HT-DEST1[CYP71BL11]

Supplementary Table 8. Overview of *N. benthamiana* expression assays for the validation of putative paralogous *CiKLS* genes.

**Assay**	**Co-infiltrated *N. benthamiana* expression vectors**
Empty vector	pEAQ-HT-DEST1[tHMGR1]; pEAQ-HT-DEST1[Empty]
CiCOS	pEAQ-HT-DEST1[tHMGR1]; pEAQ-HT-DEST1[CiGASs]; pEAQ-HT-DEST1[CYP71AV4]; pEAQ-HT-DEST1[CYP71BL3]
TpKLS	pEAQ-HT-DEST1[tHMGR1]; pEAQ-HT-DEST1[CiGASs]; pEAQ-HT-DEST1[CYP71AV4]; pEAQ-HT-DEST1[CYP71BL3]; pEAQ-HT-DEST1[TpKLS]
CYP71BZ18	pEAQ-HT-DEST1[tHMGR1]; pEAQ-HT-DEST1[CiGASs]; pEAQ-HT-DEST1[CYP71AV4]; pEAQ-HT-DEST1[CYP71BL3]; pEAQ-HT-DEST1[CYP71BZ18]
CYP71BZ19	pEAQ-HT-DEST1[tHMGR1]; pEAQ-HT-DEST1[CiGASs]; pEAQ-HT-DEST1[CYP71AV4]; pEAQ-HT-DEST1[CYP71BL3]; pEAQ-HT-DEST1[CYP71BZ19]
CYP71BZ23	pEAQ-HT-DEST1[tHMGR1]; pEAQ-HT-DEST1[CiGASs]; pEAQ-HT-DEST1[CYP71AV4]; pEAQ-HT-DEST1[CYP71BL3]; pEAQ-HT-DEST1[CYP71BZ23]
CYP71BZ20	pEAQ-HT-DEST1[tHMGR1]; pEAQ-HT-DEST1[CiGASs]; pEAQ-HT-DEST1[CYP71AV4]; pEAQ-HT-DEST1[CYP71BL3]; pEAQ-HT-DEST1[CYP71BZ20]
CYP71BZ27	pEAQ-HT-DEST1[tHMGR1]; pEAQ-HT-DEST1[CiGASs]; pEAQ-HT-DEST1[CYP71AV4]; pEAQ-HT-DEST1[CYP71BL3]; pEAQ-HT-DEST1[CYP71BZ27]
CYP71BZ25	pEAQ-HT-DEST1[tHMGR1]; pEAQ-HT-DEST1[CiGASs]; pEAQ-HT-DEST1[CYP71AV4]; pEAQ-HT-DEST1[CYP71BL3]; pEAQ-HT-DEST1[CYP71BZ25]
CYP71BZ26	pEAQ-HT-DEST1-[tHMGR1]; pEAQ-HT-DEST1[CiGASs]; pEAQ-HT-DEST1[CYP71AV4]; pEAQ-HT-DEST1[CYP71BL3]; pEAQ-HT-DEST1[CYP71BZ26]

Supplementary Table 9. Overview of *N. benthamiana* expression assays for the validation of *CYP71* genes acting downstream of kauniolide.

**Assay**	**Co-infiltrated *N. benthamiana* expression vectors**
Empty vector	pEAQ-HT-DEST1[tHMGR1]; pEAQ-HT-DEST1[Empty]
CiCOS	pEAQ-HT-DEST1[tHMGR1]; pEAQ-HT-DEST1[CiGASs]; pEAQ-HT-DEST1[CYP71AV4]; pEAQ-HT-DEST1[CYP71BL3]
[-CiCOS] CiKLS	pEAQ-HT-DEST1[tHMGR1]; pEAQ-HT-DEST1[CiGASs]; pEAQ-HT-DEST1[CYP71AV4]; pEAQ-HT-DEST1[CYP71BZ26]
CiKLS	pEAQ-HT-DEST1[tHMGR1]; pEAQ-HT-DEST1[CiGASs]; pEAQ-HT-DEST1[CYP71AV4]; pEAQ-HT-DEST1[CYP71BL3]; pEAQ-HT-DEST1[CYP71BZ26]
CYP71BZ18	pEAQ-HT-DEST1[tHMGR1]; pEAQ-HT-DEST1[CiGASs]; pEAQ-HT-DEST1[CYP71AV4]; pEAQ-HT-DEST1[CYP71BL3]; pEAQ-HT-DEST1[CYP71BZ26]; pEAQ-HT-DEST1[CYP71BZ18]
CYP71BZ19	pEAQ-HT-DEST1[tHMGR1]; pEAQ-HT-DEST1[CiGASs]; pEAQ-HT-DEST1[CYP71AV4]; pEAQ-HT-DEST1[CYP71BL3]; pEAQ-HT-DEST1[CYP71BZ26]; pEAQ-HT-DEST1[CYP71BZ19]
CYP71BZ23	pEAQ-HT-DEST1[tHMGR1]; pEAQ-HT-DEST1[CiGASs]; pEAQ-HT-DEST1[CYP71AV4]; pEAQ-HT-DEST1[CYP71BL3]; pEAQ-HT-DEST1[CYP71BZ26]; pEAQ-HT-DEST1[CYP71BZ23]
CYP71BZ20	pEAQ-HT-DEST1[tHMGR1]; pEAQ-HT-DEST1[CiGASs]; pEAQ-HT-DEST1[CYP71AV4]; pEAQ-HT-DEST1[CYP71BL3]; pEAQ-HT-DEST1[CYP71BZ26]; pEAQ-HT-DEST1[CYP71BZ20]
CYP71AV27	pEAQ-HT-DEST1[tHMGR1]; pEAQ-HT-DEST1[CiGASs]; pEAQ-HT-DEST1[CYP71AV4]; pEAQ-HT-DEST1[CYP71BL3]; pEAQ-HT-DEST1[CYP71BZ26]; pEAQ-HT-DEST1[CYP71AV27]
CYP71AV25	pEAQ-HT-DEST1[tHMGR1]; pEAQ-HT-DEST1[CiGASs]; pEAQ-HT-DEST1[CYP71AV4]; pEAQ-HT-DEST1[CYP71BL3]; pEAQ-HT-DEST1[CYP71BZ26]; pEAQ-HT-DEST1[CYP71AV25]

Supplementary Table 10. SL metabolite composition of the four SL metabolites that can be quantified using a standard (ng/mg dry weight) and relative peak area of 12 SL metabolites, for which no standard is available for quantification in leaves of diploid and tetraploid wild-type (WT) and mutant plants. Mutant genotypes (M) as well as how many individual plants were analyzed for their metabolite profile per mutant genotype are described in **Supplementary Table 5**. The mean +/- stdev is shown for the replicated measurements (biological replicates). For each individual plant, material was typically harvested for use in three technical replicates. Yet, because some regenerants containing mutations in the *CYP71* genes were very fragile after acclimatization in the greenhouse, sometimes we had to perform metabolite profiling without biological or technical repeats. This was the case for instance for M3, for which only one genotype was analyzed and only in one technical replicate and for M6, for which two genotypes were analyzed but both only in one technical replicate. Accordingly, NA indicates only one technical measurement was carried out, so no stdev could be calculated. Four compounds were quantified with reference standards: lactucin (sl11a), dihydrolactucin (sl11b), lactucopicrin (sl14a) and dihydrolactucopicrin (sl14b), while no standards were available for the other compounds: sl08a: deoxylactucin, sl08b: dihydrodeoxylactucin, sl09a: deoxylactucin glycoside, sl09b: dihydrodeoxylactucin glycoside, sl10a: deoxylactucin oxalate, sl10b: dihydrodeoxylactucin oxalate, sl12a: lactucin glycoside, sl12b: dihydrolactucin glycoside, sl13a: lactucin oxalate, sl13b: dihydrolactucin oxalate, sl15a: lactucopicrin oxalate and sl15b: dihydrolactucopicrin oxalate. Components and their sl-number refer to Figure 1. Plants overviewed between 2 thick lines are analyzed in the same run. The mutant type M6 containing a homozygous LOF mutation in the three paralogous *CiKLS* genes is indicated in red font.

M-type (ploidy level)	Sl11b ng/mg DW	Sl14b ng/mg DW	Sl11a ng/mg DW	Sl14a ng/mg DW	Sl08a	Sl09a	Sl10a	Sl10b	Sl08b	Sl09b	Sl12b	Sl13b	Sl15b	Sl12a	Sl13a	Sl15a
WT (4x)	33.81±28.15	19.58±5.71	194.55±79.60	262.71±112.34	2.49±1.06	0.15±0.07	35.83±9.84	0.75±0.22	1.64±1.56	0.49±0.21	0.99±1.00	5.41±2.45	0.59±0.09	0.05±0.03	30.03±6.70	0.99±0.37
M3 (4x)	20.25±8.40	16.75±6.82	116.32±59.92	250.80±105.04	2.20±0.47	0.16±0.01	37.01±5.99	0.72±0.13	2.02±0.23	0.57±0.10	1.15±0.18	4.22±0.69	0.52±0.10	0.01±0.00	26.04±6.32	0.83±0.15
M3 (2x)	4.79±NA	0.20±NA	39.58±NA	0.74±NA	3.52±NA	1.05±NA	1.48±NA	2.28±NA	8.10±NA	0.34±NA	3.73±NA	0.07±NA	0.92±NA	NA	NA	NA
M4 (4x)	22.43±9.52	18.26±3.63	189.05±41.97	183.61±49.65	2.37±0.40	0.11±0.01	34.24±2.07	0.69±0.03	2.48±0.10	0.96±0.12	1.41±0.12	4.30±0.05	0.43±0.05	0.01±0.00	23.83±1.87	0.60±0.12
M5 (4x)	30.79±14.87	16.48±4.74	109.73±38.79	158.79±55.88	1.13±0.28	0.15±0.03	23.64±4.10	0.40±0.06	1.38±0.31	0.24±0.07	1.23±0.12	7.20±0.96	0.46±0.04	0.02±0.01	25.82±2.12	0.53±0.07
M6 (#1) (4x)	0.00±NA	0.00±NA	0.00±NA	0.00±NA	0.00±NA	0.08±NA	0.00±NA	0.00±NA	0.00±NA	0.00±NA	0.00±NA	0.00±NA	0.00±NA	0.00±NA	0.00±NA	0.00±NA
M6 (#2)(4x)	0.00±NA	3.22±NA	0.00±NA	0.00±NA	0.00±NA	0.15±NA	0.00±NA	0.00±NA	0.00±NA	0.00±NA	0.00±NA	0.00±NA	0.00±NA	0.00±NA	0.00±NA	0.00±NA

M11 (4x)	51.24±9.71	59.74±28.46	659.21±678.28	947.25±1007.19	4.44±2.77	0.12±0.00	38.93±14.44	0.70±0.26	3.47±3.51	0.83±0.91	1.72±1.88	10.48±3.82	0.69±0.21	0.02±0.01	36.19±10.84	0.71±0.32
M13 (4x)	19.19±NA	22.38±NA	271.82±NA	271.82±NA	3.06±NA	0.19±NA	41.86±NA	0.82±NA	0.64±NA	0.45±NA	0.40±NA	4.60±NA	0.66±NA	0.08±NA	34.66±NA	1.10±NA
M20 (4x)	14.25±5.53	18.91±2.24	214.28±104.57	610.30±104.66	3.23±0.48	0.18±0.06	39.49±4.22	0.78±0.09	1.55±0.27	0.83±0.34	1.13±0.22	3.67±0.33	0.63±0.05	0.05±0.02	33.05±3.72	1.70±0.10
M30 (4x)	25.13±6.60	18.02±4.61	80.74±15.07	114.55±13.36	0.92±0.17	0.19±0.05	13.28±3.01	0.22±0.09	3.14±0.92	0.67±0.20	1.57±0.09	5.50±0.53	0.41±0.05	0.03±0.01	17.27±2.81	0.38±0.03
WT (4x)	192.66±72.14	61.23±7.35	1097.32±76.30	910.87±63.87	8.33±0.97	0.03±0.01	3.98±1.81	0.04±0.02	0.59±0.23	0.07±0.03	0.20±0.10	0.80±0.23	0.12±0.02	0.02±0.00	9.28±0.95	0.40±0.04
WT (2x)	2.84±1.70	0.04±0.02	2.10±1.46	0.04±0.02	0.15±0.11	0.02±0.01	3.09±1.42	0.20±0.15	0.82±0.29	0.98±0.83	6.40±1.34	0.07±0.10	1.07±0.19	NA	NA	NA
M5 (4x)	79.27±1.57	14.00±0.68	337.41±24.26	214.36±7.06	2.19±0.12	0.05±0.00	11.80±0.15	0.19±0.00	0.87±0.03	0.11±0.01	0.26±0.00	2.84±0.06	0.40±0.01	0.03±0.00	23.49±0.36	1.04±0.01
M11 (4x)	337.01±17.37	16.37±0.90	237.63±14.42	67.64±1.49	1.23±0.08	0.09±0.01	6.70±0.21	0.11±0.01	1.66±0.17	0.27±0.01	0.70±0.02	5.65±0.18	0.48±0.01	0.03±0.00	19.96±0.31	0.48±0.02
M12 (4x)	330.70±200.34	38.85±1.93	991.72±155.23	517.16±66.95	7.58±0.41	0.06±0.01	8.22±0.88	0.11±0.01	0.35±0.10	0.04±0.01	0.17±0.04	1.41±0.09	0.18±0.01	0.02±0.01	13.63±0.46	0.55±0.02
M14 (4x)	937.49±46.70	68.43±10.42	1821.93±287.48	1222.05±259.46	13.30±1.18	0.08±0.01	12.64±3.02	0.19±0.05	1.31±0.28	0.21±0.04	0.40±0.03	2.37±0.67	0.19±0.05	0.06±0.00	15.95±3.04	0.58±0.12
M24 (4x)	1741.28±9.64	80.46±1.57	1852.30±82.05	1116.35±48.22	13.75±0.32	0.11±0.01	7.05±0.92	0.09±0.01	1.03±0.11	0.12±0.01	0.44±0.01	1.53±0.25	0.09±0.01	0.08±0.00	11.30±1.26	0.34±0.02
M20 (4x)	126.21±0.90	62.32±3.36	1072.18±18.67	826.86±55.46	10.95±0.04	0.02±0.00	3.67±0.33	0.03±0.01	0.24±0.01	0.03±0.00	0.09±0.01	0.27±0.04	0.06±0.00	0.01±0.00	6.23±0.45	0.24±0.02
M22 (2x)	3.27±0.81	0.04±0.00	12.21±1.24	0.19±0.02	0.19±0.05	0.03±0.01	1.60±0.18	0.20±0.01	1.85±0.26	0.16±0.05	5.42±1.10	0.04±0.00	0.49±0.09	NA	NA	NA
M16 (4x)	505.62±189.96	38.19±10.22	1114.38±344.02	711.83±320.49	8.76±2.45	0.07±0.02	16.36±5.24	0.26±0.10	0.54±0.13	0.07±0.02	0.31±0.11	2.53±0.97	0.29±0.10	0.04±0.01	22.67±5.41	0.89±0.22
M46 (2x)	7.52±0.05	0.03±0.00	3.75±0.36	0.05±0.00	0.06±0.01	0.01±0.00	3.30±0.04	0.12±0.01	0.60±0.04	0.51±0.02	8.96±0.05	0.02±0.00	1.02±0.05	NA	NA	NA
M46 (4x)	754.49±21.20	67.44±2.37	1626.70±91.86	738.91±36.79	10.09±0.30	0.07±0.01	8.69±0.61	0.12±0.02	0.90±0.03	0.14±0.01	0.44±0.03	2.48±0.08	0.20±0.01	0.05±0.00	14.75±0.46	0.46±0.03
WT (4x)	157.80±47.30	53.57±8.70	1346.72±232.38	960.78±264.44	8.12±1.8	0.02±0.01	4.95±1.79	0.14±0.05	0.56±0.17	0.06±0.04	0.17±0.09	0.79±0.24	0.12±0.03	0.01±0.00	9.77±0.82	0.45±0.07
WT (2x)	2.25±1.19	0.03±0.01	2.50±1.78	0.11±0.05	0.16±0.06	0.01±0.00	2.89±1.33	0.21±0.12	0.99±0.21	0.88±0.81	6.14±1.15	0.06±0.08	0.89±0.19	NA	NA	NA
M34 (2x)	3.78±0.23	0.02±0.00	2.60±0.57	0.07±0.00	0.05±0.01	0.00±0.00	1.25±0.07	0.02±0.01	0.36±0.12	0.49±0.07	4.65±0.25	0.00±0.00	0.64±0.05	NA	NA	NA
M33 (4x)	51.86±0.76	32.37±1.53	1043.62±39.04	1014.65±30.09	9.90±0.60	0.02±0.00	10.54±1.33	0.19±0.00	0.11±0.01	0.01±0.00	0.03±0.01	0.52±0.03	0.12±0.00	0.00±0.00	10.70±1.00	0.68±0.02
M38 (4x)	82.16±15.45	31.32±7.17	895.60±268.95	728.25±316.39	9.24±2.04	0.02±0.01	13.92±2.99	0.24±0.08	0.16±0.11	0.02±0.02	0.05±0.02	0.97±0.37	0.19±0.06	0.00±0.00	13.54±2.50	0.81±0.18
M39 (4x)	157.42±22.00	41.08±5.42	1120.12±244.60	1343.30±399.89	8.97±2.03	0.03±0.01	8.35±6.12	0.22±0.06	0.29±0.18	0.04±0.04	0.09±0.06	0.60±0.45	0.11±0.07	0.01±0.00	9.29±5.71	0.52±0.26

M40 (4x)	74.68±4.13	30.75±2.83	952.45±82.45	1089.63±197.82	6.46±0.22	0.03±0.00	5.79±1.62	0.12±0.02	0.06±0.02	0.01±0.00	0.04±0.01	0.51±0.13	0.12±0.03	0.01±0.00	10.04±1.76	0.70±0.11
M42 (4x)	74.54±NA	8.92±NA	363.45±NA	72.66±NA	0.84±NA	0.02±NA	3.72±NA	0.09±NA	0.22±NA	0.04±NA	0.21±NA	2.47±NA	0.22±NA	0.02±NA	18.08±NA	0.56±NA
M43 (4x)	74.50±27.25	37.76±2.41	1048.17±57.30	1216.22±40.46	9.75±0.35	0.02±0.01	8.00±0.72	0.14±0.01	0.09±0.01	0.01±0.00	0.03±0.01	0.36±0.01	0.12±0.01	0.00±0.00	7.94±0.47	0.66±0.02
M27 (4x)	98.96±70.72	30.49±23.15	592.54±531.10	574.33±567.23	6.19±5.61	0.03±0.02	15.62±8.05	0.26±0.12	1.11±1.46	0.14±0.18	0.19±0.14	1.66±0.80	0.26±0.12	0.01±0.01	13.55±3.54	0.72±0.18
M25 (4x)	134.31±1.72	59.68±4.56	1148.55±154.35	1519.83±156.75	11.39±0.45	0.01±0.00	2.08±0.44	0.24±0.01	0.44±0.05	0.03±0.00	0.10±0.01	0.09±0.02	0.04±0.01	0.01±0.00	2.28±0.41	0.18±0.02
